# Presbycusis: Pathology, Signal Pathways, and Therapeutic Strategy

**DOI:** 10.1002/advs.202410413

**Published:** 2025-05-11

**Authors:** Xiaoxu Zhao, Tian Shen, Shengda Cao, Ziyi Liu, Wendu Pang, Meixuan Li, Jingjing Liu, Wen Li, Yunhao Wu, Chengcheng Liu, Ming Xia, Xiaolong Fu, Cheng Cheng

**Affiliations:** ^1^ Medical Science and Technology Innovation Center Shandong First Medical University & Shandong Academy of Medical Sciences Jinan Shandong 250117 China; ^2^ Department of Otolaryngology‐Head & Neck Surgery West China Hospital Sichuan University Chengdu 610072 China; ^3^ Department of Otorhinolaryngology Qilu Hospital of Shandong University NHC Key Laboratory of Otorhinolaryngology (Shandong University) Jinan Shandong 250117 China; ^4^ Institute of Brain Science and Brain‐inspired Research Shandong Provincial Hospital Shandong First Medical University & Shandong Academy of Medical Sciences Jinan Shandong 250117 China; ^5^ Department of Otolaryngology The First Affiliated Hospital of Wenzhou Medical University Wenzhou Zhejiang 325000 China; ^6^ College of Life Science Shandong Normal University Jinan Shandong 250117 China; ^7^ Department of Central Laboratory Shandong Provincial Hospital Affiliated to Shandong First Medical University Jinan Shandong 250117 China; ^8^ Department of Otolaryngology Shandong Provincial Hospital Affiliated to Shandong First Medical University Department of Otolaryngology Shandong Provincial Hospital, Shandong University NHC Key Laboratory of Otorhinolaryngology Jinan Shandong 250117 China; ^9^ Shandong Provincial Hospital Medical Science and Technology Innovation Center School of Clinical and Basic Medical Sciences Shandong First Medical University & Shandong Academy of Medical Sciences Jinan Shandong 250117 China

**Keywords:** pathology, presbycusis, stria vascularis, single‐cell transcriptomic therapeutic strategy

## Abstract

Presbycusis, also known as age‐related hearing loss (ARHL), is a progressive auditory impairment and ranks among the most prevalent sensory disorders in the elderly population. It is primarily caused by damage to hair cells, degeneration of spiral ganglion neurons, and atrophy of the stria vascularis, which are integral components of the cochlea. While extensive research has been devoted to hair cells and spiral ganglion neurons, this review focuses on the critical role of the stria vascularis in ARHL. The primary function of the stria vascularis is to maintain the endocochlear potential, which is essential for hearing. This review presents an overview of the research concerning the stria vascularis in ARHL, particularly focusing on the application of single‐cell transcriptomics to elucidate its role. Furthermore, this review explores relevant signaling pathways and therapeutic strategies for ARHL. By enhancing the understanding of the stria vascularis in ARHL, this review aims to pave the way for personalized treatment and improved strategies for protection and prevention.

## Graphical abstract

In ARHL, the stria vascularis, acting as a cochlear battery, gradually loses its ability to maintain the endocochlear potential, leading to impaired hair cell function and progressive hearing loss. Single‐cell sequencing reveals age‐related cellular changes in the cochlea, providing insights into the underlying mechanisms of aging and potential therapeutic strategies.

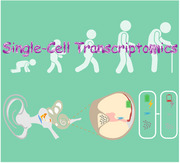

## 1 Introduction

**Figure 1:**
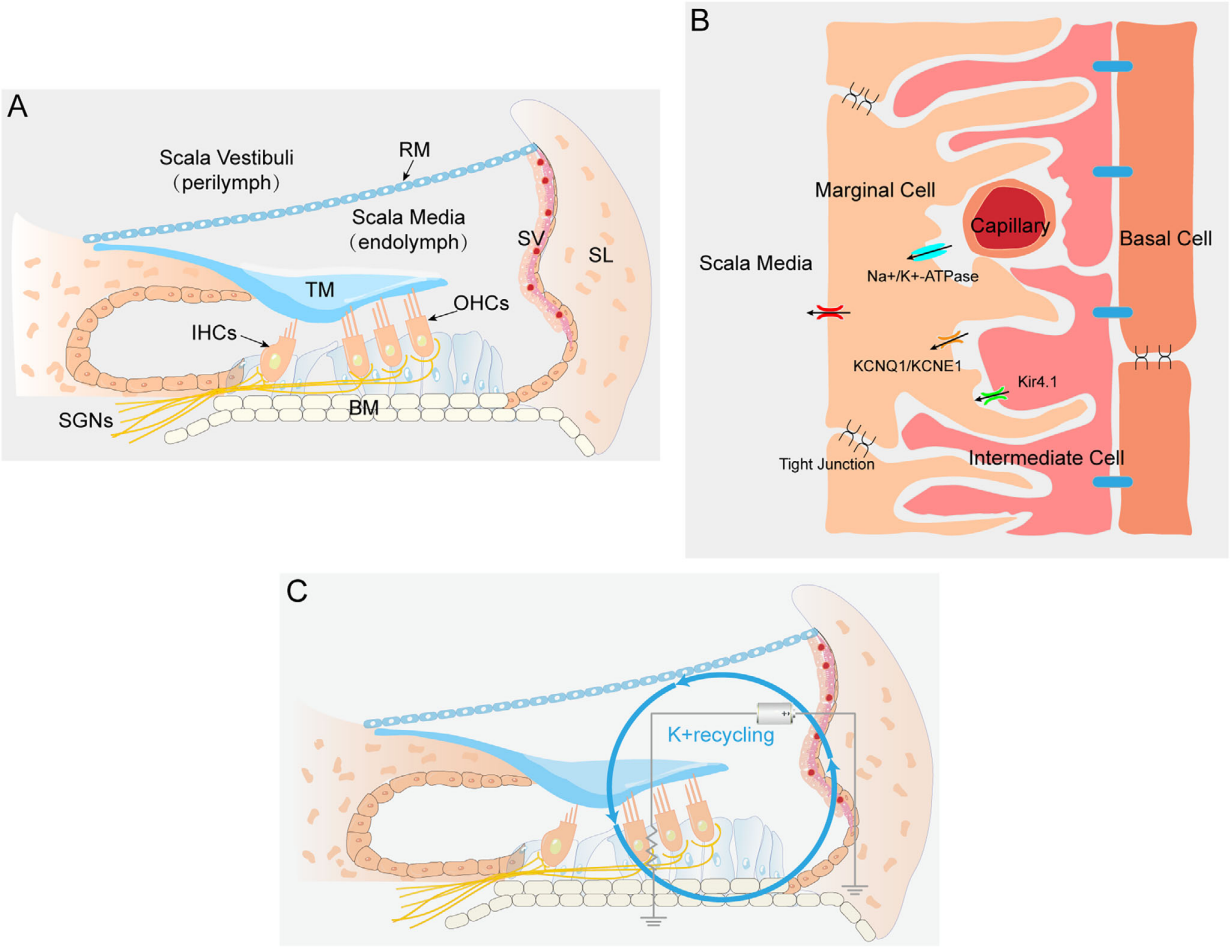
A) A cross section of the cochlea. The mechanical‐gated K^+^ channels in the stereocilia of HCs are stimulated by sound waves, allowing the influx of K^+^ and depolarization of the HCs. As the HCs depolarize, K^+^ exits the cell, is absorbed by the fibrocytes of the spiral ligament, and is transported to the SV, returning to the endolymph. B) Diagram of the SV. C) The SV is the cochlear battery.

Presbycusis, commonly referred to as age‐related hearing loss (ARHL), encompasses various types of hearing impairment observed in older individuals.^[^ ^1^ ^]^ The World Health Organization predicts that by 2025, with the aging population's growth, ≈1 billion people aged 65 and older will suffer from hearing loss.^[^ ^2^ ^]^ ARHL is characterized by a decline in hearing sensitivity, reduced capacity to comprehend language in noisy environments, slower processing of auditory information, and impaired sound localization abilities.^[^ ^3^, ^4^ ^]^ These factors result in challenges during one‐on‐one conversations, impacting the enjoyment of music and participation in social interactions, with the severity of these consequences being directly related to the extent of hearing impairment.^[^ ^5^ ^]^ The cochlea, an important organ for sensing sound, is mainly composed of hair cells (HCs), stria vascularis (SV), and spiral ganglion neurons (SGNs) (**Figure** **1**A). Based on the pathological alterations of the cochlear components, Schuknecht et al. classified ARHL into four types according to the structural composition of the cochlea: sensory (loss of HCs), neural (loss of cochlear neurons), metabolic (atrophy of SV), and cochlear conductive (changes in the conduction or resonance of the cochlear duct).^[^ ^6^ ^]^ Although an increasing amount of research on ARHL has focused on the sensory hair cells and the ribbon synapses that form connections between HCs and SGNs, it is essential to acknowledge that there may be other potentially interacting mechanisms due to the high heterogeneity of ARHL.^[^ ^7^, ^8^ ^]^ This review focuses on recent research on SV, including the application of newer technologies, such as single‐cell transcriptomics in SV, aiming to offer novel insights into the study of metabolic ARHL.

Obtaining cochlear tissue samples presents a significant challenge due to their location within dense temporal bone, necessitating invasive surgery that raises ethical concerns and limits the feasibility of direct experimental manipulations. Additionally, ARHL has been associated with environmental factors, particularly noise exposure.^[^ ^9^ ^]^ However, accurately measuring an individual's noise exposure throughout their lifetime is impossible, complicating the distinction between naturally occurring pathological ARHL due to aging and ARHL induced by noise exposure. These limitations can largely be overcome when using animal models, which can be studied in a controlled environment and experimentally manipulated, thus providing more accurate data on the direct causes of ARHL. Studies involving animal models, specifically the Mongolian gerbil (*Meriones unguiculatus*), have shown that when gerbils are exposed to continuous noise, they exhibit variable HCs loss, particularly outer hair cells.^[^ ^10^ ^]^ In contrast, quiet‐aged gerbils show only minimal HCs loss, suggesting that hearing loss in older gerbils may be more related to metabolic ARHL.^[^ ^10^, ^11^ ^]^ Synaptopathy, characterized by the deterioration of auditory nerve fibers connected to inner hair cells, occurs in quiet‐aged gerbils but does not lead to a decrease in hearing thresholds.^[^ ^12^, ^13^, ^14^ ^]^ Therefore, the development of ARHL in quiet‐aged gerbils presents a prime opportunity to investigate the effects of strial dysfunction.^[^ ^14^ ^]^ Among various mouse models, the inbred C57BL/6J mouse strain is controversial because it carries a mutation in the Cdh23 gene, which encodes a hair cell stereociliary protein believed to accelerate HCs loss.^[^ ^9^ ^]^ Interestingly, the endocochlear potential (EP) of the C57BL/6J mouse strain did not significantly change as the aged.^[^ ^15^ ^]^ Moreover, there is no definitive evidence indicating that HCs loss affects the SV function.^[^ ^16^, ^17^ ^]^ Thus, the C57BL/6J mouse strain is also a worthy ARHL mouse model when studying the effects of aging on SV function, including ion transport, altered pigmentation, inflammatory response, and vascular atrophy.

Aging is a progressive process characterized at the molecular level by the loss and damage of genetic information and at the cellular level by the decline or apoptosis of cellular function, as well as the weakening of intercellular connections.^[^ ^18^ ^]^ These changes subsequently impact organ structure and function, culminating in the overall aging of the organism.^[^ ^18^ ^]^ The aging of the cochlea is intricately linked to this broader aging process. For instance, genetic instability or leakage within HCs can trigger inflammatory responses, leading to damage in HCs as individuals age.^[^ ^19^ ^]^ Furthermore, the aging process weakens the cellular connections between HCs and SGNs, impairing the transmission of auditory signals to the central nervous system.^[^ ^20^, ^21^ ^]^ Additionally, age‐related changes in specific signaling pathways may result in either upregulation or downregulation, triggering cellular apoptosis.^[^ ^22^, ^23^ ^]^ Herranen et al. demonstrated that mesencephalic astrocyte‐derived neurotrophic factor (MANF) plays a protective role for outer hair cells in the cochlea against endoplasmic reticulum (ER) stress‐mediated cell death. This protective function is crucial in certain mouse strains susceptible to early‐onset ARHL, such as C57BL/6J and CD‐1.^[^ ^24^ ^]^ The hallmark features of ARHL include a gradual decline in hearing ability with advancing age, primarily affecting high‐frequency hearing.^[^ ^4^ ^]^ Cellular aging, including the degeneration of HCs and other age‐related changes such as vascular atrophy, represents a significant process.^[^ ^25^, ^26^ ^]^ These changes contribute to the progressive decline in cochlear function associated with ARHL. Notably, the sensitivity and severity of ARHL vary significantly among individuals, influenced by factors such as genetics, environmental exposures, and lifestyle choices.^[^ ^27^, ^28^, ^29^, ^30^, ^31^ ^]^ While the structural and cellular anatomy of the cochlea has been well described, high‐resolution molecular analyses are still required to unravel the specific mechanisms through which age‐related molecular changes contribute to hearing loss.^[^ ^32^, ^33^, ^34^, ^35^, ^36^ ^]^ Therefore, investigating the interconnections between cells and structures in the cochlea, along with the alterations in cellular signaling pathways, is crucial for developing strategies to prevent and alleviate ARHL. Recent advancements, including single‐cell RNA sequencing (scRNA‐seq), have offered valuable insights by identifying transcriptional signatures associated with aging and related diseases across multiple organs and heterogeneous tissues.^[^ ^37^, ^38^, ^39^, ^40^ ^]^ This review will explore ARHL from the perspective of the interrelationships among SV, HCs, and SGNs, focusing on the pathological changes and signaling pathway alterations in these cell types during the progression of ARHL, alongside potential therapeutic strategies.

## 2 Pathology

When sound enters the ear, it is transmitted through the outer and middle ear before reaching the inner ear.^[^ ^41^ ^]^ In the inner ear, the SV regulates the ionic balance of the inner ear fluid, ensuring an optimal auditory environment.^[^ ^42^, ^43^ ^]^ This fluid plays a crucial role in hearing by supporting the HCs within the cochlea.^[^ ^42^, ^43^ ^]^ The HCs consist of inner hair cells (IHCs) and outer hair cells (OHCs), which play distinct roles; specifically, OHCs amplify sound signals, while IHCs transduce sound waves into neural impulses.^[^ ^19^, ^44^ ^]^ These neural impulses are subsequently transmitted by SGNs, a network of neurons within the cochlea.^[^ ^20^ ^]^ The SGNs relay the auditory signals to the brain's auditory center, enabling the perception and interpretation of sound.^[^ ^20^ ^]^

With aging, the SV, HCs, and SGNs all undergo degeneration, which contributes to presbycusis. However, these components do not age simultaneously; their aging processes occur asynchronously and are driven by distinct underlying mechanisms.^[^ ^45^, ^46^ ^]^ Therefore, it is essential to examine each key component and the pathological changes associated with their respective aging processes individually. Additionally, adopting an integrated perspective is crucial for understanding their interconnections and mutual influences throughout the aging process.

### 2.1 Stria Vascularis

The SV, a highly vascularized tissue located in the lateral wall of the cochlea, plays a crucial role in maintaining cochlear homeostasis by regulating ions and ensuring the unique ionic composition of cochlear fluids.^[^ ^47^, ^48^ ^]^ The SV is composed of three main cell types: marginal, intermediate, and basal cells.^[^ ^49^ ^]^ Marginal cells form a monolayer derived from the cochlear duct epithelium, which directly faces the endolymph (Figure 1A,B).^[^ ^50^ ^]^ These cells are abundant in K^+^ transporters, including the Na^+^/K^+^‐ATPase pump and the Na^+^‐K^+^‐2Cl^‐^ cotransporter 1, which enable the SV to maintain K^+^ transport.^[^ ^51^ ^]^ Intermediate cells, situated between the marginal and basal cell layers, contribute to melanin production, provide protection against oxidative damage, and K^+^ transport within the SV.^[^ ^52^ ^]^ Basal cells, located adjacent to the spiral ligament, are interconnected by gap junctions, forming a barrier between the interatrial space and the perilymph.^[^ ^53^ ^]^ The tight intercellular adhesion between marginal and basal cells is integral to mediating ion transport within the cochlea.^[^ ^54^ ^]^

The cochlea comprises three tubular structures: the scala media, scala tympani, and scala vestibuli (Figure 1A). The scala tympani and scala vestibuli are filled with perilymph, which has an ionic composition similar to that of typical extracellular fluids. In contrast, the scala media contains a unique fluid known as endolymph. The endolymph is characterized by 150 mm K^+^, 2 mm Na+, 20 µm Ca2+, and an EP of ≈+80 mV relative to blood plasma and perilymph.^[^ ^55^, ^56^ ^]^ When sound waves stimulate HCs, K^+^ ion channels on the stereocilia open, allowing K^+^ to enter and depolarize the HCs.^[^ ^57^ ^]^ Subsequently, K^+^ exits through basolateral channels and reaches the lateral cochlear wall.^[^ ^58^, ^59^ ^]^ This process, known as K^+^ cycling, involves the continuous movement of K^+^ ions from the endolymph to the perilymph and back to the endolymph (Figure 1C). K+ cycling ensures a high concentration of K+ in the endolymph, thereby maintaining the EP and supporting cochlear function.^[^ ^47^ ^]^ Although Wang et al. demonstrated that OHCs can adapt transducer operation point in response to an acutely lowered EP, recovering nearly full cochlear amplification,^[^ ^60^ ^]^ the long‐term effect of EP reduction due to aging remains unclear. Kaur et al. found that a flatter audiometric shape was associated with greater strial atrophy in humans.^[^ ^61^ ^]^ However, audiometric assessments are subjective and can be influenced by the technician's skill level and proficiency. Currently, no noninvasive, objective method exists to directly measure EP in humans, making it challenging to distinguish between metabolic and sensory ARHL. However, in animal models, EP and SV function can be measured invasively using microelectrodes placed within the cochlea.^[^ ^62^ ^]^ Ding et al. employed qPCR and immunofluorescence techniques to compare the expression of ion channel‐related proteins in the SV of 3‐week‐old and 1.5‐year‐old CBA/CaJ mice, revealing a significant decline in protein expression in aging mice.^[^ ^63^ ^]^ This reduction was associated with cochlear potential imbalance and subsequent hearing loss.^[^ ^63^, ^64^, ^65^ ^]^ Interestingly, Wu et al. challenged this perspective by analyzing 120 human inner ears obtained during autopsy, concluding that HCs death preceded SV atrophy in the progression of ARHL.^[^ ^66^ ^]^ However, this study relied primarily on histopathology and lacked the means to assess the EP or ion circulation within the cochlea, leaving important questions unanswered.

Oxidative stress, driven by reactive oxygen species (ROS) and mitochondrial oxidative damage, significantly contributes to aging processes.^[^ ^4^, ^19^, ^67^, ^68^, ^69^, ^70^ ^]^ Tan et al. found that the knockout of *Fus1*, a mitochondrial protein encoded by a nuclear gene, induces ARHL in mice.^[^ ^71^ ^]^ In these models, abnormalities in the SGNs and SV were characterized by increased mitochondrial ROS, decreased mitochondrial membrane potential, disrupted calcium signaling, and impaired mitochondrial fusion.^[^ ^71^ ^]^ Yamane et al. observed that acoustic overstimulation elevates cochlear ROS,^[^ ^72^ ^]^ while Ohlemiller et al. reported that ROS initially accumulates in the marginal cells of the SV.^[^ ^73^ ^]^ Within 1–2 h of noise exposure, ROS levels increased fourfold.^[^ ^74^ ^]^ In addition to mitochondria, peroxisomes are also closely associated with ROS production and regulation.^[^ ^22^ ^]^ However, their roles in ARHL, particularly in the SV, remain poorly understood. Although animal models facilitate numerous studies on the SV, including the detection of EP loss,^[^ ^9^, ^55^, ^56^, ^57^, ^60^ ^]^ ROS accumulation,^[^ ^75^ ^]^ ion channel knockouts,^[^ ^76^ ^]^ and the effects of furosemide application,^[^ ^77^, ^78^ ^]^ dynamic and systematic research at the molecular level remains limited. This may be due to the fact that even in animal models, the small size and volume of the SV make it challenging to study deeply at the molecular level.

To investigate the cellular and molecular mechanisms underlying ARHL, we conducted dynamic single‐cell transcriptomic profiling of the cochlea in C57BL/6J mice at 1, 2, 5, 12, and 15 months of age. To uncover aging‐related transcriptional perturbations, we first performed an overall coefficient of variation (CV) analysis. This analysis revealed an age‐related increase in CV, with significantly higher values in mice at 2, 5, 12, and 15 months compared to those at 1 month, peaking at 5 months.^[^ ^45^ ^]^ When comparing all cochlear cells at 5 months to those at 1‐month, intermediate cells within the SV exhibited higher CV values. Next, we identified aging‐associated positively correlated genes within the intermediate cells, uncovering several known genes linked to aging and hearing loss. For instance, *Smad5*, an effector of the TGF‐β signaling pathway,^[^ ^79^ ^]^ is associated with cochlear cell fibrosis and inflammation. Pairwise differentially expressed gene (PDEG) analysis revealed significant age‐related changes in gene expression at 5 months, with pronounced upregulation and downregulation of genes in basal cells. By 12 months, substantial aging‐related gene changes were observed in intermediate and basal cells. Notably, the highest number of PDEGs was identified in intermediate cells, characterized by persistent upregulation of PDEGs through the 15th month. These changes were linked to increased protein folding, modulation of cell death, and reductions in rhythmic processes and cell‐cell junction assembly, indicating a gradual and progressive decline in the function of intermediate cells with age. In contrast, HCs and SGNs, two crucial cochlear cell types, did not show a significant increase in PDEGs. This suggests that cellular aging in the cochlea initiates within the SV. To further investigate gene expression dynamics, we analyzed dynamic differentially expressed genes (DDEGs) across multiple time points.^[^ ^80^ ^]^ Based on the total number of DDEGs, the top five cell types included intermediate cells, chondrocytes, macrophages, Schwann cells, and B cells, highlighting their susceptibility to aging. We identified a negative correlation between intracellular Na^+^ homeostasis, melanin biosynthesis, and gap junction assembly with aging. As these processes are integral to the function of intermediate cells,^[^ ^81^, ^82^ ^]^ this finding suggests that the cellular functions of the intermediate layer may progressively deteriorate during the aging process. More importantly, we observed an upregulation of unfolded protein response (UPR) related pathways in intermediate cells with aging. Concurrently, apoptotic pathways in the SV also exhibited age‐associated upregulation. This implies that adaptive UPR mechanisms may be insufficient to mitigate sustained endoplasmic reticulum stress, ultimately contributing to the irreversibility of cochlear aging. These findings underscore the critical involvement of stria microvasculature in the age‐related degeneration of the SV. Additionally, Samaha et al. utilized single‐nucleus RNA‐Seq datasets to determine that both pro‐inflammatory and anti‐inflammatory factors are mainly enriched in the SV.^[^ ^83^ ^]^ Major pro‐inflammatory cytokines expressed include *Tnfrsf21, Ifnar2, Nfkb1, Mif, Il1rap, Il6ra, Il6st*, and *Il17re*, while key cytokines and cytokine receptor genes exhibiting significant expression include *Tgfb2, Il10rb, Il15ra, Tgfbr2, Tgfbr3, Il15, Jak1, Stat2, Stat3*, and *Stat5b*.^[^ ^83^ ^]^ However, the precise impact of inflammatory factors on the SV in ARHL remains unclear and requires further investigation.

### 2.2 Sensory Hair Cells

In the human cochlear structure, it is estimated that there are ≈3,500 IHCs and ≈12,000 OHCs.^[^ ^32^ ^]^ IHCs exhibit a vase‐like morphology, with their stereocilia arranged in a U‐shaped pattern on the apical surface. These cells are embedded in and grow from the basement membrane, forming synaptic connections with 4 to 13 type I afferent nerve fibers.^[^ ^36^ ^]^ In contrast, OHCs are cylindrical and organized into three parallel rows along the basilar membrane. The stereocilia on OHCs are arranged in a V‐shaped or W‐shaped pattern, creating a staircase‐like array. When sound vibrations reach the inner ear, the amplification of these vibrations by OHCs causes the bending of their stereocilia, leading to the opening of transduction channels. This process stimulates the SGNs, which transmit sound frequencies to the nervous system in a tonotopic manner, thereby creating a frequency map.^[^ ^84^ ^]^

Over time, the accumulation of internal and external insults leads to damage to HCs, contributing to ARHL in the elderly. Several case‐based histopathological studies of human temporal bone have provided evidence regarding the timing of HCs degeneration.^[^ ^32^, ^41^, ^85^, ^86^ ^]^ HCs degeneration has been observed in infants and newborns as young as a few hours old,^[^ ^32^, ^86^ ^]^ and in some cases, degeneration may commence in utero.^[^ ^86^ ^]^ However, since this degeneration is also observed in the cochleae of adults with normal hearing, it is considered part of a normal developmental process.^[^ ^86^ ^]^ Based on available anatomical evidence, it is hypothesized that OHCs degeneration may begin in the basal regions of the cochlea and then progress toward the middle,^[^ ^87^, ^88^ ^]^ a pattern also observed in the SV.^[^ ^42^ ^]^ Notably, the age‐related loss of OHCs is more pronounced than that of IHCs.^[^ ^32^, ^89^, ^90^ ^]^ A study of autopsy samples from 20 subjects aged 0 to 89 found that individuals over 60 experienced an average loss of OHCs between 30–40% across the audiometric frequency range of 0.25–8.0 kHz. In contrast, the average loss of IHCs across all frequencies seldom exceeded 15% in any age group.^[^ ^87^ ^]^ High‐frequency hearing loss is a typical feature in the audiograms of ARHL patients. The loss of OHCs in the basal turn of the cochlea is thought to significantly contribute to age‐related threshold shifts. In contrast, the loss of OHCs in the apical region does not manifest in the audiogram. One plausible explanation is that the “motors” of OHCs have a reduced contribution to cochlear amplification at the cochlear apex compared to the cochlear base.^[^ ^87^ ^]^

Interestingly, synaptic loss in IHCs occurs in the cochlea of aging mice before any measurable changes in auditory thresholds or HCs counts are observed.^[^ ^91^, ^92^ ^]^ This process is further accelerated in animals that have previously experienced noise‐induced acute synaptopathy at an early age.^[^ ^93^ ^]^ Redundancy, a common feature of many biological systems, allows for the loss of a few cells without significant functionality. Remarkably, even with the loss of up to 80% of IHCs, only a minor shift in pure tone thresholds is observed when measured behaviorally.^[^ ^94^ ^]^ Nevertheless, the loss of IHCs may still affect listening in noisy environments, even when thresholds remain within the normal range.^[^ ^95^ ^]^

Noise, genetic defects, and ototoxic drugs can further exacerbate damage to sensory hair cells. Studies comparing cochlear samples from individuals with a history of noise exposure, often occupational, to ‘normal‐aging’ controls reveal that noise exposure primarily aggravates OHCs loss in the high‐frequency (basal turn) region of the cochlea. In contrast, IHCs remain unaffected by both low and high‐frequency noise exposure.^[^ ^87^ ^]^ This suggests that while the loss of apical OHCs is associated with aging, the basal loss is partly attributable to noise exposure, which is difficult to avoid in many human environments. Given that ARHL is closely associated with environmental factors, its genetic basis cannot be fully uncovered through traditional family‐based linkage analysis. Previous attempts to elucidate the genetic susceptibility of ARHL through cohort‐based analyses have had limited reproducibility. This may be due to the fact that genome‐wide associated studies (GWAS) require thousands of samples, and the collection of hearing data is constrained by the availability of well‐trained otologists, controlled quiet environments, and substantial time commitments. Nevertheless, GWAS has contributed to identifying candidate genes that help elucidate the genetic composition of ARHL. Among the strongest candidate genes is *GRM7*, which encodes the glutamate metabotropic receptor 7 (*GRM7*). This finding has been replicated in several independent cohorts, although not in all populations tested.^[^ ^96^, ^97^, ^98^, ^99^ ^]^ GRM7 is a G‐protein coupled receptor activated by L‐glutamate, and its activation is associated with reduced neurotransmitter release. As *GRM7* is expressed in both HCs and SGNs, variations in *GRM7* may lead to increased glutamate release at the synapse between IHCs and auditory neurons, thus contributing to susceptibility to ARHL.^[^ ^96^ ^]^ Another gene closely associated with ARHL is *TRIOBP*, which encodes TRIO and F‐actin binding protein, a regulator of the actin cytoskeleton.^[^ ^100^ ^]^ Characterization of *Triobp* isoform‐specific knockout mice has revealed a role for TRIOBP in the formation of cochlear hair cell stereocilia rootlets, which are essential for providing mechanical rigidity to the stereocilia bundle.^[^ ^101^ ^]^

The relationship between drugs that may cause hearing loss and ARHL is complex and influenced by several factors. Ototoxic drugs, including aminoglycosides, cisplatin, carboplatin, loop diuretics (such as furosemide and bumetanide), and non‐steroidal anti‐inflammatory drugs (NSAIDs), can damage the delicate structures of the inner ear, particularly the HCs and auditory nerve fibers, both of which are essential for hearing. Aminoglycosides typically cause initial damage to OHCs in the cochlea's basal high‐frequency region, with cumulative dosing leading to progressive damage toward the apex.^[^ ^102^, ^103^ ^]^ In contrast, IHCs are generally more resistant to aminoglycosides.^[^ ^102^ ^]^ Animal studies have shown that cisplatin administration leads to degeneration in the organ of Corti, characterized by the partial or complete loss of OHCs, sporadic loss of IHCs, and damage to the SV.^[^ ^104^ ^]^ The extent to which ototoxic drugs accelerate the aging process of cochlear cells and their role in the pathophysiology of ARHL remains unclear. However, recent population‐based longitudinal studies conducted over a decade have begun to provide insights into these interactions.^[^ ^105^ ^]^ Research has indicated that ototoxicity can exacerbate the effects of aging, potentially worsening hearing loss beyond what is typically seen with age alone.^[^ ^105^ ^]^ It is essential to consider that older adults, who often rely on ototoxic drugs to manage chronic conditions, are more vulnerable to their adverse effects. Age‐related declines in renal function and pre‐existing alterations in the ear further increase their susceptibility to the harmful impacts of these medications.

HCs are highly energy‐dependent, and mitochondrial dysfunction severely impacts their function.^[^ ^4^, ^70^ ^]^ Research conducted by Fischel‐Ghodsian et al. demonstrates that patients diagnosed with ARHL exhibit a significant accumulation of mutations in the mitochondrially‐encoded cytochrome oxidase II gene, in contrast to individuals without ARHL.^[^ ^106^ ^]^ Furthermore, studies of human temporal bones have identified a frequent 4977 base pair deletion in mtDNA, known as the ‘common deletion,’ which is associated with the cytochrome c oxidase subunit III gene in cochlear tissues of ARHL patients. Additionally, three other novel deletions within the same gene have also been observed.^[^ ^107^, ^108^ ^]^ In aged OHCs, mitochondrial abnormalities are evident, including absence or disorganized cristae, reduced mitochondrial numbers, diminished membrane potential, and lower calcium levels.^[^ ^109^, ^110^ ^]^ These findings suggest that mitochondrial dysfunction and reduced energy production play a critical role in the onset of ARHL, potentially preceding the loss of OHCs.

### 2.3 Spiral Ganglion Neurons

In addition to the age‐related decrease in EP in the inner ear, the cochlea and auditory nerve in elderly individuals undergo further physiological changes.^[^ ^27^, ^111^, ^112^ ^]^ One notable change is the loss of auditory nerve function, which is evidenced by an increased threshold for the CAP of the auditory nerve. When measuring the electrical signals (action potentials) in the auditory nerves of older individuals, a decrease in signal strength (reduced amplitudes) is frequently observed. This reduction likely indicates that nerve activity is no longer occurring in a well‐coordinated or synchronized manner. The precise pathological basis for this asynchronous activity remains unclear; however, it may involve the synapse between auditory nerve fibers and IHCs, degeneration of SGNs, and a reduction in EP of the inner ear. Consequently, distinguishing between auditory nerve dysfunctions caused by diminished EP and those due to spiral ganglion cell degeneration remains a significant challenge. In contrast to age‐related central degeneration in the auditory nervous system, asynchronous activity in the auditory nerve may play a more prominent role in the development of ARHL.

Human SGNs are classified into two types: ≈95% are type I, while 5–10% are type II afferent neurons. Type I and type II SGNs, which innervate the IHCs and OHCs, respectively, exhibit significant differences in their connectivity and function, which are also evident at the molecular level.^[^ ^113^, ^114^ ^]^ Type I SGNs, characterized by their myelination, are primarily responsible for encoding auditory signals as traditionally understood.^[^ ^115^ ^]^ In contrast, type II SGNs, which lack myelination, may play a role in mediating auditory pain and nociception.^[^ ^116^ ^]^

Tuj1, a marker for neural differentiation, can distinguish between type I and type II SGNs.^[^ ^117^ ^]^ Previous research has shown a decrease in both overall cell density and the density of Tuj1‐positive pan‐neurons in the modiolus, particularly in the basal turn of the cochlea, in 15‐month‐old mice.^[^ ^45^ ^]^ Focusing on type I SGNs, three subtypes have been identified based on their distinct gene expression profiles: type IA (Chchd10+), type IB (Calb+), and type IC (*Pou4f1*+).^[^ ^118^ ^]^ Studies have characterized their distribution patterns in the apex, middle, and base regions of the cochlea, revealing age‐related changes in their distribution. The observed gradient distribution of various subtypes along the frequency axis suggests that these SGNs play different but complementary roles in processing sound frequencies. Specifically, subtypes Ia and Ib SGNs may be more involved in processing low frequencies, while subtype Ic likely contributes to high‐frequency processing.^[^ ^119^ ^]^ As previous studies have shown, the overall density of SGNs declines with age. However, only subtype Ic SGNs showed a selective decline, decreasing from 30% at 32 weeks to 20% at 64 weeks, and further declining to 11% at 108 weeks. Meanwhile, the proportion of Ia and Ib SGNs increased. This selective decline of subtype Ic SGNs makes them particularly susceptible to ARHL.^[^ ^120^ ^]^

The initial belief was that the degeneration of SGNs occurs more slowly than the loss of HCs, suggesting that the presence of HCs is a necessary condition for the survival of SGNs.^[^ ^32^, ^41^ ^]^ However, recent studies have challenged this viewpoint, indicating that the loss of SGNs can occur independently of HCs degeneration.^[^ ^121^ ^]^ To reassess the necessity of IHCs for neuron survival, a study utilizing a high‐affinity thiamine transporter protein (*Slc19a2*) knockout mouse model was conducted. In this model, mice maintained on a regular (thiamine‐rich) diet exhibited normal cochlear structure and function. However, dietary thiamine restriction led to rapid and widespread loss of IHCs within 10 days. Notably, it was observed that SGNs can survive for several months following the loss of IHCs, indicating that IHCs are not essential for the maintenance of SGNs in the adult cochlea.^[^ ^122^ ^]^ Conversely, the innervation of the inner ear is critically dependent on neurotrophins such as Ntf3 and BDNF.^[^ ^123^ ^]^ Mutant mice lacking both neurotrophins exhibit an initially normal development of HCs. However, by postnatal day 10, nearly all innervation is lost, and within 3 weeks, complete denervation results in a near‐total loss of OHCs and many IHCs by 4 months. Mutants retaining one allele of either neurotrophin show partial innervation loss, leading to a slower progression of HCs loss. Notably, HCs in these mutants disappear in a base‐to‐apex pattern, similar to the progression observed after carboplatin ototoxicity. Denervation of vestibular sensory epithelia also results in variable outcomes, ranging from aberrant HCs resembling those in the organ of Corti to near‐normal HCs. These findings suggest a significant role for innervation in the long‐term maintenance of HCs.^[^ ^123^ ^]^

Human temporal bone specimens devoid of apparent otologic diseases and exhibiting no significant loss of inner and outer hair cells were utilized to evaluate age‐related primary degeneration of cochlear neurons.^[^ ^85^ ^]^ The count of SGNs in these specimens demonstrated a mean decline rate of 100 cells per year throughout life.^[^ ^85^ ^]^ These findings indicate that the loss of SGNs in human presbycusis is now recognized as more than a secondary consequence of HCs loss. The increasing acknowledgment of primary SGNs loss as a significant factor in ARHL challenges previous assumptions. SGNs are bipolar neurons characterized by a peripheral axon directed toward the organ of Corti and a central axon projecting to the cochlear nucleus. Notably, the peripheral axon undergoes more pronounced age‐related degeneration than the central axon.^[^ ^124^ ^]^ A reduction in the peripheral axon can be observed even in patients who retain normal populations of HCs and SGNs.^[^ ^125^, ^126^ ^]^ It is clear that many SGNs, along with their central axons, survive for years after the degeneration of their peripheral axons. Recent studies indicate that the age‐related loss rate of peripheral axons exceeds that of IHCs.^[^ ^87^ ^]^ These findings suggest that a significant number of SGNs in the aging ear become disconnected from their target HCs.^[^ ^87^ ^]^ While this disconnection may not affect audiogram thresholds, it could contribute to difficulties in hearing in noisy environments for older people.^[^ ^87^ ^]^ These discoveries enhance our understanding of the role of SGNs peripheral axons in presbycusis. In scRNA‐seq studies of the mouse cochlea, we observed a consistent upregulation of the *Sncb* gene in SGNs with aging.^[^ ^45^ ^]^ The *Sncb* gene encodes a member of a small family of proteins that inhibit phospholipase D2, which is abundant in neurofibrillary lesions in Alzheimer's disease and has been implicated in triggering oxidative stress and inflammatory responses in the aged retina.^[^ ^127^ ^]^ These findings suggest that as SGNs experience increased oxidative stress and inflammatory responses due to aging, SGNs injury may exacerbate ARHL.

## 3 Signaling Pathways in ARHL

**Figure 2:**
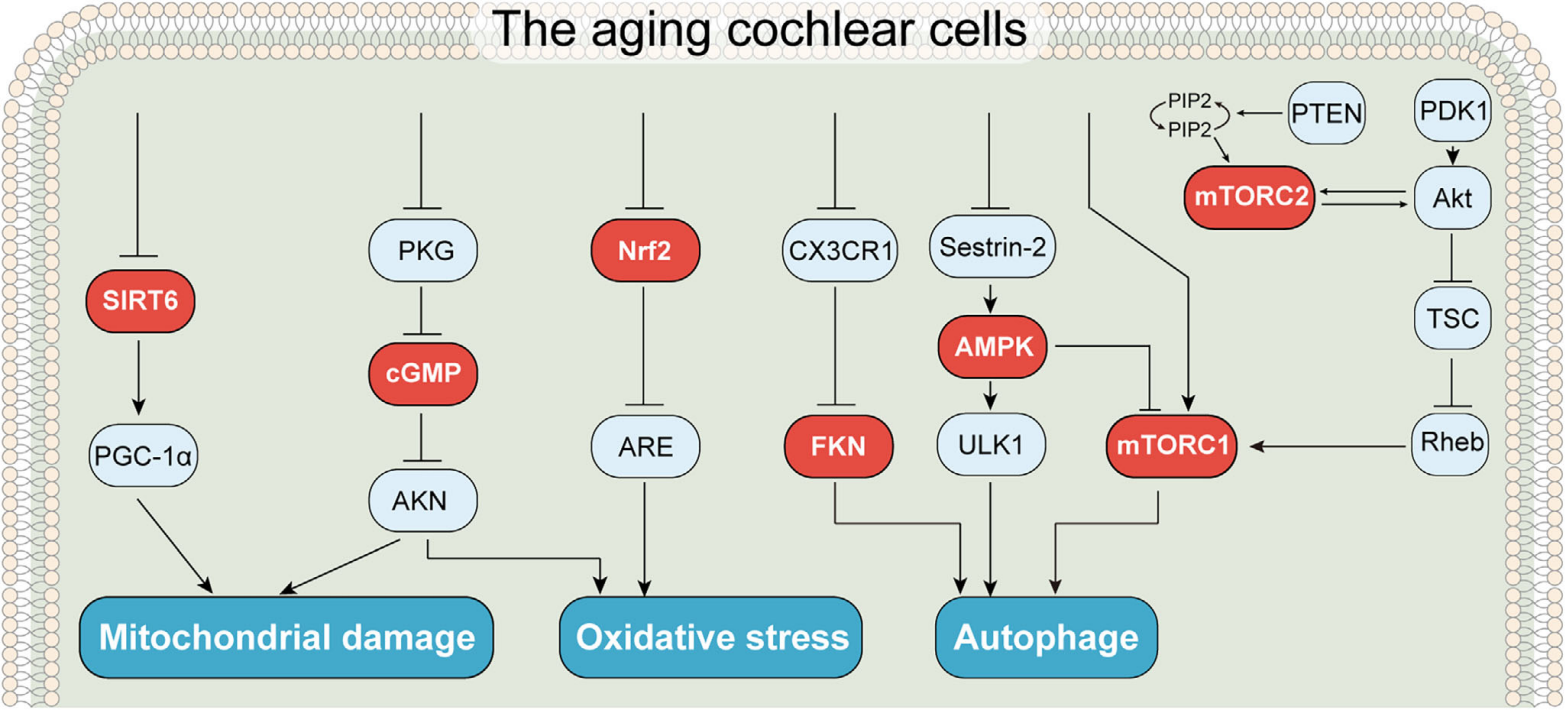
Signaling pathways in the aging cochlea. (i) The expression of SIRT1 dramatically declines in the cochlea and auditory cortex of aged mice, resulting in the downregulation of PGC1α and subsequent mitochondrial dysfunction; (ii) Age‐related reduction in PKG and decline in cGMP signaling lead to the disappearance of protective effect by ANP on the neurogenesis of SGNs, and further result in mitochondrial dysfunction and oxidative stress, which in turn associated with ARHL; (iii) Oxidative stress caused by aging impair the function of the Nrf2/ARE signaling pathway. In addition, the weakening of the Nrf2/ARE signaling pathway leads to a decrease in the expression of antioxidant enzymes and detoxifying enzymes, which further results in oxidative stress in cells; (iv) Age‐related reduction in expression of CX3CR1, leading to the blocking the signal pathway and further result in the release of inflammatory cytokines, spiral ganglion damage and ARHL; (v) Sestrin 2 can suppress mTORC1 by activating AMPK, thereby reducing the accumulation of ROS; (vi) The role of mTORC signaling in aging. Phosphoinositide 3‐kinase (PI3K) catalyzes the phosphorylation of the lipid phosphatidylinositol 4,5‐bisphosphate (PIP2), resulting in the conversion of PIP2 to phosphatidylinositol 3,4,5‐trisphosphate (PIP3). PIP3 serves as a binding site for Akt (protein kinase B or PKB) and 3‐phosphoinositide‐dependent protein kinase‐1 (PDK1). This positioning allows Akt to be close to PDK1, facilitating Akt Thr308 site phosphorylation and resulting in partial activation. The Ser473 site is phosphorylated by mTORC2, triggering the full enzymatic activity of Akt. Another pathway of downstream Akt targets is mTORC1, a central regulator of cell growth, and Akt acts as a positive regulator of mTORC1.

ARHL is a complex condition influenced by various factors, including genetics, environmental exposures (such as noise), physiological changes associated with aging, and other potential ototoxic elements. Through a comprehensive investigation into the impact of these factors on the cochlea, we have summarized that at the molecular level, several contributors to ARHL may share common signaling pathways (**Figure** **2**).^[^ ^18^, ^30^ ^]^ These pathways involve various cell types in the cochlea, primarily regulating apoptotic and metabolic pathways.

In addition to the abovementioned factors, other ototoxic elements, including certain drugs and chemical substances, may exacerbate hearing loss.^[^ ^81^ ^]^ These substances likely mediate their ototoxic effects through various signaling pathways. Understanding the distinct and overlapping signaling pathways involved in ARHL is crucial for developing more effective treatment and prevention strategies. In‐depth research into the activation and interactions of these signaling pathways will provide a robust foundation for developing targeted interventions, ultimately enhancing therapeutic outcomes. Here, we aim to provide a summary and introduction to the diverse signaling pathways implicated in the activation and progression of ARHL.

### 3.1 PI13K/Akt/mTOR Signaling Pathway

The mammalian target of rapamycin (mTOR) is a serine/threonine kinase that exists in two distinct complexes.^[^ ^128^ ^]^ mTORC1, which is sensitive to the drug rapamycin, primarily regulates cell growth, protein synthesis, and autophagy and includes key components such as mTOR, Raptor, and mLST8.^[^ ^128^, ^129^, ^130^ ^]^ Conversely, mTORC2, which exhibits lower sensitivity to rapamycin, regulates cell survival and cytoskeletal organization, comprising mTOR, Rictor, Sin1, and mLST8.^[^ ^128^, ^129^, ^130^ ^]^ The activation of Akt inhibits the tuberous sclerosis complex (TSC), which consists of TSC1, TSC2, and Tre2–Bub2–Cdc16‐1 domain family member 7 (TBC1D7). This inhibition alleviates the TSC complex's inhibitory effect on Ras homolog enriched in the brain (RHEB), ultimately activating mTORC1.^[^ ^131^, ^132^ ^]^

A study demonstrated that mice treated with rapamycin starting at 4 months of age experienced reduced age‐related OHCs loss in the basal half of the cochlea at 22 months, compared to untreated counterparts.^[^ ^133^ ^]^ However, when rapamycin was administered at 14 months of age, it reduced ARHL but did not prevent OHCs loss.^[^ ^134^ ^]^ To investigate the role of mTORC1 signaling in ARHL, we observed an elevation in p‐S6 and p‐P70S6K, both of which are downstream targets of mTORC1, within the sensory epithelium of the ARHL mouse model.^[^ ^22^ ^]^ Subsequently, we created raptor‐conditional knockout (cKO) mice to inactivate mTORC1 signaling; these mice retained more HCs in the aging inner ear, effectively preventing ARHL.^[^ ^22^ ^]^ In contrast, *Tsc1*‐cKO mice, which exhibited sustained mTORC1 activation, showed early‐onset cochlear HCs death, leading to accelerated hearing loss.^[^ ^22^ ^]^ Administering rapamycin along with the antioxidant N‐acetylcysteine effectively protected the HCs of *Tsc1*‐cKO mice from damage.^[^ ^22^ ^]^ These findings suggest that mTOR signaling may play a crucial role in the progression of ARHL.

The AMPK and TOR signaling pathways are interconnected, functioning as counteractive mechanisms that detect energy availability and regulate cellular growth.^[^ ^135^ ^]^ AMPK inhibits mTORC1 activity primarily through the phosphorylation of TSC2, which activates the TSC complex and suppresses RHEB, thereby inhibiting mTORC1 activity.^[^ ^135^ ^]^ Under energy‐deprived conditions, such as elevated AMP/ATP or ADP/ATP ratios, AMPK inhibits mTORC1 to conserve energy. Additionally, Sestrin 2 suppresses mTORC1 by activating AMPK, which reduces the accumulation of ROS.^[^ ^136^ ^]^ A study revealed a decline in Sestrin 2 expression in the aging cochlea, and Sestrin 2 KO mice exhibited extensive OHCs loss compared to C57BL/6J control mice.^[^ ^137^ ^]^

Recent research has demonstrated that the overexpression of uncoupling protein 2 (UCP2) leads to ATP depletion and excessive ROS generation, which activates AMPKα and intensifies apoptosis of IHCs, primarily in the apical and middle turns of the cochlea.^[^ ^138^ ^]^ Conversely, reducing AMPK activity has been shown to mitigate apoptosis within the cochlea. For instance, employing AMPKα1 KO as a rescue strategy for Tg‐mtTFB1 mice (a model of mitochondrial deafness) resulted in improvements in ABR wave I, EP, and IHCs function, alongside the normalization of SGNs, IHCs synapses morphology, and enhanced survival of OHCs.^[^ ^139^ ^]^ Despite these insights, research on the role of AMPK in ARHL remains limited, necessitating further studies to elucidate the molecular mechanisms through which AMPK influences ARHL. Future research could explore whether the effects of AMPK on ARHL operate in conjunction with or independently of mTOR and autophagy.

### 3.2 SIRT1 Signaling Pathway

SIRT1, a member of the sirtuin family, is an NAD^+^‐dependent deacetylase that regulates various aging‐related signaling pathways, including NF‐κB, AMPK, mTOR, P53, PGC1α, and FoxOs.^[^ ^140^ ^]^ It achieves this regulation by deacetylating key proteins, which delays cellular senescence. SIRT1 plays a pivotal role in energy homeostasis by modulating glucose and lipid metabolism, primarily through the deacetylation of PGC‐1α, a master regulator of mitochondrial biogenesis, oxidative phosphorylation, and mitochondrial dynamics.^[^ ^141^ ^]^

Several miRNAs can regulate SIRT1 expression by binding to the 3' UTR of its mRNA, which can either lead to its degradation or inhibit its translation. Research has indicated a significant decline in SIRT1 expression in the aging mouse cochlea and auditory cortex, a deficiency that is linked to the expression of miR‐34a in these regions during aging.^[^ ^142^, ^143^ ^]^ Furthermore, studies have demonstrated that either overexpression of SIRT1 or knockdown of miR‐34a mitigates age‐related cochlear HCs loss and hearing loss in C57BL/6 mice.^[^ ^144^ ^]^ Similarly, Xue et al. showed that overexpression of miR‐29b results in HCs loss and mitochondrial dysfunction through downregulation of the SIRT1/PGC‐1α signaling pathway with aging.^[^ ^145^ ^]^ Notably, the rs1894720 polymorphism in the myocardial infarction‐associated transcript (MIAT) gene has been shown to increase susceptibility to ARHL by altering the miR‐29b/SIRT1/PGC‐1α signaling pathway.^[^ ^146^ ^]^

LncRNA H19 has been shown to regulate SIRT1 expression via miR‐653‐5p in vitro, thereby influencing the viability of HEI‐OC1 cells.^[^ ^147^ ^]^ Pang et al. elucidated that SIRT1 protects HCs and delays ARHL in C57BL/6 mice through the activation of autophagy.^[^ ^148^ ^]^ SIRT1 is activated in response to cellular stress, particularly during energy depletion or caloric restriction, resulting in increased levels of NAD^+^. It can also be activated by various small molecules, including resveratrol and thymoquinone. Kim et al. demonstrated that β‐lapachone (β‐lap), a plant‐derived metabolite, enhances NAD^+^ levels through quinone oxidoreductase 1 (NQO1) activity, which increases SIRT1 activity and ameliorates age‐related hearing impairment.^[^ ^149^ ^]^ Furthermore, resveratrol, thymoquinone, and ubiquinol‐10 have been found to reduce HCs loss and delay ARHL in mouse models by activating SIRT1.^[^ ^143^, ^144^, ^148^, ^150^, ^151^ ^]^ Recent studies also suggest that cocoa polyphenol extract inhibits the senescence of auditory cells through the induction of both SIRT1 and SIRT3 in vitro, underscoring the protective role of SIRT1 upregulation against hearing loss.

In contrast to these findings, Han et al. observed that 12‐month‐old SIRT1^+/−^ mice maintained normal hearing at middle and high frequencies, whereas age‐matched wild‐type mice exhibited typical ARHL. This observation suggests that a deficiency in SIRT1 may protect HCs and delay ARHL in C57BL/6 mice.^[^ ^152^ ^]^ These contrasting results underscore the complex role of SIRT1 in ARHL, and the underlying mechanisms explaining these divergent findings remain unclear. Given SIRT1's involvement in a wide array of aging‐related pathways, further investigation into its mechanisms in aging and ARHL could provide valuable insights to reconcile these conflicting results.

SIRT3, another sirtuin family member, plays a crucial role in regulating mitochondrial function and metabolism, particularly in fatty acid oxidation and the electron transport chain. Caloric restriction has been shown to upregulate SIRT3, which enhances the mitochondrial antioxidant defense system, thereby delaying the onset of ARHL in mice.^[^ ^153^ ^]^

### 3.3 Nrf2/ARE Signaling Pathway

Nrf2 is a redox‐sensitive transcription factor that plays a crucial role in regulating oxidative stress.^[^ ^154^ ^]^ Upon synthesis, Nrf2 translocates to the cell nucleus, where it induces the expression of various antioxidant response element (ARE)‐dependent genes, including heme oxygenase‐1 (HO‐1), superoxide dismutase (SOD), catalase (CAT), and glutathione synthetase (GS).^[^ ^154^ ^]^

A population‐based study demonstrated a statistically significant correlation (p‐value < 4 × 10^−2^) between two genes, PRKCE and TGFB1, which are associated with the NRF2 pathway.^[^ ^155^ ^]^ This finding suggests that oxidative stress, along with the regulation of gene expression via the NRF2‐ARE pathway, plays a crucial role in the pathogenesis of hearing loss, particularly in a mouse model of deafness. These results support the hypothesis that NRF2 pathways are essential for maintaining hearing function, with their impairment potentially contributing significantly to the development of ARHL (Figure 2).^[^ ^155^ ^]^

Previous research also shows that resveratrol can reduce ROS levels in SGNs and supporting cells by activating the Nrf2‐ARE signaling pathway. This activation promotes the proliferation and differentiation of SGNs, thereby improving neural hearing loss in mice.^[^ ^156^ ^]^ The weakening of the Nrf2/ARE signaling pathway, often observed with aging, results in a decrease in the expression of antioxidant and detoxifying enzymes, rendering cells more susceptible to oxidative stress. This oxidative damage further impairs the Nrf2/ARE signaling pathway, creating a vicious cycle of progressive degeneration.

### 3.4 Fractalkin/CX3CR1 Signaling Pathway

FKN (Fractalkine) is a unique transmembrane protein expressed by neurons and endothelial cells, associated with age‐dependent changes in the cochlea.^[^ ^157^ ^]^ What makes FKN distinctive is its exclusive signaling through the receptor CX3CR1, which is expressed on certain leukocytes, including monocytes, macrophages, and microglia.^[^ ^158^, ^159^ ^]^ The migration of local macrophage and the infiltration of bone marrow‐derived peripheral blood macrophage into the damaged cochlea occur through multiple signaling cascades mediated by specific chemical signals released from damaged sensory and non‐sensory cells in the cochlea. One such pathway is the FKN/ CX3CR1 signaling pathway, which serves as a direct communication channel between macrophage and the IHCs and SGNs in the damaged cochlea.^[^ ^160^ ^]^

Research has shown that the disruption of FKN signaling, resulting from the genetic absence of the CX3CR1 receptor on macrophages, increases the death of SGNs two months after selective ablation of HCs, compared to animals with intact FKN signaling.^[^ ^161^ ^]^ Therefore, age‐related changes in CX3CR1 expression can block this signaling pathway, leading to further damage to SGNs and contributing to the development of ARHL. This suggests that HCs, SV, and SGNs may share the same signaling pathway during aging in the cochlea (Figure 2).

### 3.5 ANP/NPRA/cGMP/PKG Signaling Pathway

Cyclic guanosine monophosphate (cGMP) is a critical secondary messenger that mediates various biological functions through three primary effector molecules: cGMP‐dependent protein kinase, cGMP‐gated ion channels, and cGMP‐regulated phosphodiesterase isoforms. Pathways involving cGMP play a crucial role in regulating neuronal development and synaptic plasticity.^[^ ^162^ ^]^ Atrial natriuretic peptide (ANP), brain natriuretic peptide (BNP), and C‐type natriuretic peptide (CNP) are structurally related peptides belonging to the natriuretic peptide (NP) family. These peptides are widely distributed throughout the central nervous system (CNS) of mammals, particularly in peripheral sensory organs such as the cochlear ganglion.^[^ ^163^, ^164^ ^]^

Research suggests that ANP plays an important role in the neurogenesis of SGNs during inner ear development. It influences key processes such as the outgrowth, elongation, and branching of SGNs neurites. The manipulation of cGMP levels and the activation of PKG via ANP and its receptor signaling represent promising therapeutic strategies to enhance SGNs neurite regeneration and support SGNs survival.^[^ ^165^ ^]^ However, age‐related reduction in PKG levels and declines in cGMP signaling impair the protective effects of ANP on SGNs neurogenesis, which is associated with ARHL.

## 4 Strategies for Prevention and Treatment

**Figure 3:**
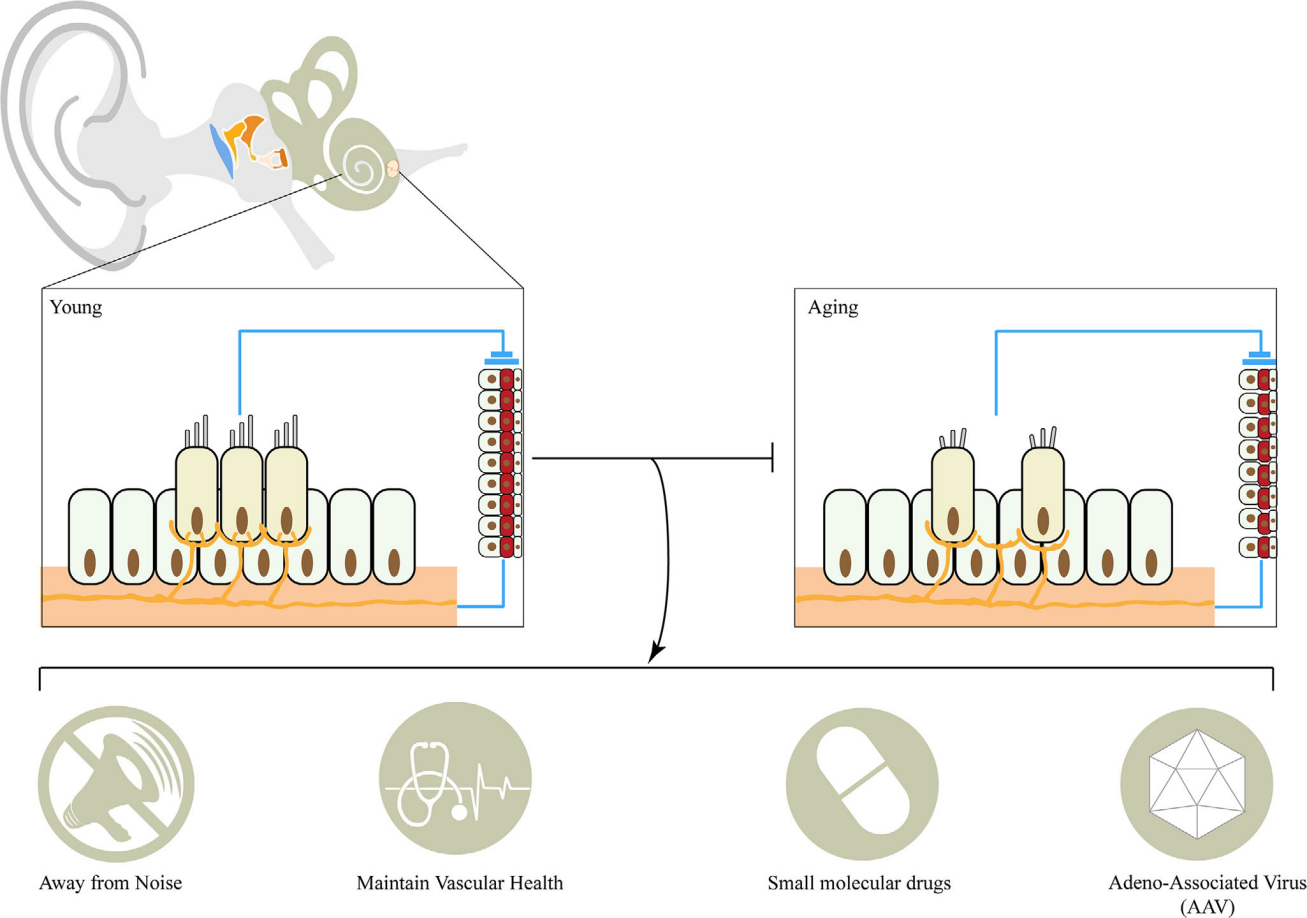
Current therapeutic strategies:(i) Avoid situations with excessive noise; (ii) Maintain cardiovascular health; (iii) Pharmacological intervention using small molecule drugs; (iv) Deliver normal gene transcripts via viral vectors. The symbols used in the figures are sourced from Freepik.

In the preceding sections, we have discussed the causes underlying the onset and progression of presbycusis, including its pathology and molecular pathways. In this section, we will briefly introduce preventive and therapeutic strategies for managing presbycusis (**Figure** **3**).

Currently, mild to moderate ARHL is primarily treated with hearing aids, while severe cases necessitate cochlear implants, assuming there are sufficient surviving neuronal cells. In either case, it is not possible to restore ‘natural’ hearing levels. ARHL typically results from a combination of damage to HCs, neuronal injury, and damage to the SV.

In contrast to certain avian species, mammals lack the inherent ability to spontaneously regenerate damaged HCs.^[^ ^166^ ^]^ In recent years, the scientific community has focused on elucidating the underlying mechanisms of this process to develop effective therapeutic strategies for stimulating HCs regeneration in mammals. Two primary strategies have emerged at the forefront of these efforts: 1) the direct trans‐differentiation of supporting cells into HCs through the exogenous expression of atonal homolog 1 (ATOH1), and 2) mitotic HCs regeneration, in which supporting cells first undergo mitosis before differentiating into HCs, achieved by inactivating cell‐cycle inhibitors, such as the cyclin‐dependent kinase inhibitor p27Kip1.^[^ ^167^ ^]^ Recently, Qi et al. conducted a review emphasizing the synergistic regulation of multiple signaling pathways, which collectively trigger HCs regeneration.^[^ ^168^ ^]^

Following the promising results of HCs regeneration in animal studies utilizing ATOH1, the CGF166 clinical trial employed an adenovirus to deliver the human *ATOH1* gene into the cochleae of patients with hearing loss.^[^ ^169^ ^]^ This trial aimed to evaluate the safety, tolerability, and potential efficacy of CGF166 as a therapeutic option for hearing loss. Partial Phase I/II clinical trial data were made available online on October 8, 2021. However, ATOH1 alone has not proven sufficient to regenerate lost HCs. Yamoah et al. suggested that the reciprocal interaction between *Sox2* and *Atoh1* may enhance regenerative potential.^[^ ^170^ ^]^ HCs regeneration represent a paradigm‐shifting strategy for addressing ARHL. This approach holds the potential to alleviate hearing impairment and fundamentally reverse a widespread sensory deficit encountered in the aging population.

As discussed earlier, cochlear synaptopathy, also known as ‘hidden hearing loss,’ has garnered significant attention in ARHL research due to its profound impact on speech perception in acoustically challenging environments despite normal hearing thresholds in conventional audiometric assessments. Gene therapy aims to address the genetic causes of hearing loss, with adeno‐associated virus (AAV)‐mediated gene therapy showing particular promise. Specifically, AAV serotype 1 carrying the human OTOF transgene (AAV1‐hOTOF) has been investigated as a potential treatment for autosomal recessive deafness 9, demonstrating both safety and efficacy in clinical trials involving children with confirmed OTOF mutations.^[^ ^171^, ^172^ ^]^ This approach involves the direct delivery of the human otoferlin coding sequence to the cochlea, overcoming the size limitation of single AAV vectors, which cannot accommodate the full‐length otoferlin coding sequence. The successful restoration of auditory function in OTOF knockout mouse models has paved the way for human clinical trials, marking a significant advancement in the treatment of genetic hearing loss. These trials aim to evaluate dose‐limiting toxicity and improvements in auditory function, offering a promising new treatment option that could greatly enhance the quality of life for individuals with genetic hearing loss. Furthermore, several studies have shown that AAV‐mediated delivery of exogenous BDNF and NT3 can independently and effectively reverse cochlear synaptopathy induced by noise exposure.^[^ ^173^, ^174^ ^]^

Future research should explore the neuroprotective roles of neurotrophins in mitigating the degenerative progression of SGNs and synapses associated with ARHL. Furthermore, it is crucial to conduct in‐depth investigations into the molecular pathways involved in ARHL, as well as a comprehensive assessment of the effects of neurotrophic factors at various stages of cochlear development. This approach aims to maximize their protective benefits while minimizing potential toxic effects on cochlear cells.

Recent studies have underscored the connection between mTORC1 signaling and redox balance, revealing these alterations in mTORC1 signaling influence ROS levels.^[^ ^175^, ^176^ ^]^ Rapamycin, a well‐known mTOR inhibitor, appears to delay intrinsic aging in mammals, significantly extending their lifespan.^[^ ^177^ ^]^ In vivo studies have shown that treating HCs lacking *Tsc1* with rapamycin, in conjunction with the antioxidant N‐acetylcysteine, rescues HCs from injury. Liu et al. found that rapamycin can improve ARHL by enhancing autophagy in SGNs as well.^[^ ^178^ ^]^ This approach effectively reduced mTORC1 activity in both HCs and SGNs, showing promise in preventing ARHL.^[^ ^22^ ^]^ However, high concentrations of rapamycin have been observed to induce rapid deafness in mice.^[^ ^23^ ^]^ Consequently, rapamycin may not be an ideal pharmacological agent for treating ARHL, as high doses of sirolimus decrease mTORC2 activity. Therefore, there is a pressing need to develop specific and efficient inhibitors of mTORC1 that do not affect mTORC2 to prevent ARHL.

Another concern regarding rapamycin is its potential side effects, particularly its immunosuppressive effects. Encouragingly, several novel rapamycin analogs, including ATP‐competitive mTOR inhibitors, have shown remarkably reduced side effects in lymphocytes compared to rapamycin.^[^ ^22^ ^]^ Choo et al. investigated the effects of atorvastatin, a statin drug, on ARHL through drug repurposing, revealing that atorvastatin significantly preserved hearing in aging mice, particularly in the early stages of ARHL.^[^ ^179^ ^]^ This protective effect was attributed to the drug's capacity to maintain mitochondrial integrity and enhance antioxidant activity by activating the HSF1/Sirt1 signaling pathway, which is crucial for reducing oxidative stress and apoptosis in cochlear cells.^[^ ^179^ ^]^

Halonen et al. further investigated the therapeutic potential of aldosterone, a mineralocorticoid hormone, in decelerating the progression of ARHL.^[^ ^180^ ^]^ Their findings indicated that long‐term systemic treatment with aldosterone enhanced both behavioral and electrophysiological measures of hearing in middle‐aged mice. The mechanism underlying this protective effect was linked to the upregulation of NKCC1 protein expression in the cochlear lateral wall, which is essential for maintaining the EP and for auditory transduction. However, there remains a lack of clarity regarding the optimal dosage and duration of these treatments, and insufficient clinical data is available. Therefore, further investigation into their mechanisms of action is crucial to enhance both safety and efficacy.

The gene *Gabra1*, which plays a role in neuronal processes, exhibits differential expression with aging in the organ of Corti and SGNs. This gene encodes the alpha1 subunit of the ionotropic GABAA receptor, which has been identified in SGNs and is involved in modulating afferent neurotransmission through the release of GABA from the lateral efferent system.^[^ ^181^ ^]^ Drugs, including ethchlorvynol, methohexital, zaleplon, and brexanolone, that inhibit the GABAA alpha1 subunit may potentially treat ARHL by reducing lateral efferent inhibition and thereby enhancing afferent neurotransmission.

Currently, therapeutic options for the SVs are limited. Improving blood circulation, consuming a healthy diet, reducing alcohol consumption, and ceasing smoking are important lifestyle changes.^[^ ^182^ ^]^ Preventing and treating chronic diseases, as well as exercising caution with ototoxic medications, are also crucial for preventing further cochlear damage.^[^ ^31^ ^]^ Recent single‐cell transcriptome studies have mapped the dynamic changes in cochlear aging in mice, revealing the cellular and molecular regulatory mechanisms, along with key time points in cochlear function decline.^[^ ^45^ ^]^ Greater changes in gene expression levels with age were observed in marginal cells in the SV, suggesting that marginal cell‐related genes may serve as potential regulators of cochlear aging damage. Nevertheless, much remains to be learned regarding the prevention and treatment of SVs degeneration.

## 5 Conclusion

As life expectancy continues to rise, presbycusis has become increasingly prevalent, depriving older individuals of crucial sensory input and significantly impacting their quality of life. Recent evidence suggests that hearing loss may serve as an early indicator of dementia and could contribute to its development.^[^ ^183^ ^]^ While numerous studies have focused on ARHL, much of the research often limits itself to specific components of the cochlea, such as HCs, SV, and SGNs. Currently, there is a dearth of research on the overall coordination of the cochlea. Studies have shown that aging‐related signaling pathways are also consistent across different cochlear components. With the continuous advancement of spatial transcriptomics and single‐cell sequencing technologies, exploring the molecular correlations between HCs, SV, and SGNs may provide a novel approach to preventing and treating ARHL.

Currently, the primary strategies for preventing presbycusis include avoiding noise exposure, maintaining a balanced diet, and engaging in regular exercise to promote auditory health. By exploring the interconnected pathways involved in ARHL, it becomes possible to develop more precise and targeted pharmaceutical treatments. For individuals already experiencing presbycusis, hearing aids are considered a viable treatment option. Concurrently, numerous studies are being conducted on gene and cell therapies.^[^ ^184^, ^185^, ^186^, ^187^ ^]^ However, many unresolved challenges persist in these fields. These challenges include enhancing the specificity of AAV vectors for targeting the appropriate cells, improving the virus's resistance to the immune system, stabilizing gene expression through modifications to the AAV expression elements, and refining delivery systems for small molecule drugs or viruses.

This article ultimately concludes by exploring the impact of various cochlear structures on ARHL, with a particular emphasis on the role of the SV. Furthermore, it provides an overview of the latest treatment approaches and molecular signaling pathways associated with ARHL, offering a novel perspective on the investigation and therapy of ARHL.

## Conflict of Interest

The authors declare no conflict of interest.

## Author Contributions

X.Z., T.S., S.C., and Z.L. contributed equally to this work.

## References

[1] G. A. Gates , J. H. Mills , Lancet. 2005, 366, 1111. 16182900 10.1016/S0140-6736(05)67423-5

[2] S. Chadha , K. Kamenov , A. Cieza , B World Health Organ. 2021, 99, 242. 10.2471/BLT.21.285643 PMC8085630 33953438

[3] G. M. Sprinzl , H. Riechelmann , Gerontology. 2010, 56, 351. 20090297 10.1159/000275062

[4] T. Yamasoba , S. Someya , C. Yamada , R. Weindruch , T. A. Prolla , M. Tanokura , Hear. Res. 2007, 226, 185. 16870370 10.1016/j.heares.2006.06.004

[5] H. Hu , Y. Ma , B. Ye , Q. Wang , T. Yang , J. Lv , J. Shi , H. Wu , M. Xiang , Neuroscience. 2017, 341, 1. 27867060 10.1016/j.neuroscience.2016.11.013

[6] H. F. Schuknecht , M. R. Gacek , Ann Otol Rhinol Laryngol. 1993, 102, 1. 10.1177/00034894931020S101 8420477

[7] M. A. Eckert , K. C. Harris , H. N. Lang , M. A. Lewis , R. A. Schmiedt , B. A. Schulte , K. P. Steel , K. I. Vaden , J. R. Dubno , Hear. Res. 2021, 402, 108109. 33189490 10.1016/j.heares.2020.108109 PMC7927149

[8] K. I. Vaden Jr. , M. A. Eckert , L. J. Matthews , R. A. Schmiedt , J. R. Dubno , J. Assoc. Res. Otolaryngol. 2022, 23, 253. 35064426 10.1007/s10162-021-00826-y PMC8964894

[9] K. K. Ohlemiller , J. Assoc. Res. Otolaryngol. 2002, 3, 444. 12486599 10.1007/s10162-002-2041-y PMC3202445

[10] R. A. Schmiedt , J. H. Mills , J. C. Adams , Hear. Res. 1990, 45, 221. 2358415 10.1016/0378-5955(90)90122-6

[11] B. I. Tarnowski , R. A. Schmiedt , L. I. Hellstrom , F. S. Lee , J. C. Adams , Hear. Res. 1991, 54, 123. 1917712 10.1016/0378-5955(91)90142-v

[12] F. Steenken , A. N. Heeringa , R. Beutelmann , L. Zhang , S. Bovee , G. M. Klump , C. Koppl , Neurobiol. Aging 2021, 108, 133. 34601244 10.1016/j.neurobiolaging.2021.08.019

[13] S. Bovee , G. M. Klump , S. J. Pyott , C. Sielaff , C. Koppl , Int. J. Mol. Sci. 2024, 25, 2738. 38473985 10.3390/ijms25052738 PMC10931817

[14] A. N. Heeringa , C. Koppl , Hear. Res. 2019, 376, 111. 30862414 10.1016/j.heares.2019.02.015

[15] K. K. Ohlemiller , Brain Res. 2009, 1277, 70. 19285967 10.1016/j.brainres.2009.02.079 PMC2792931

[16] D. O. Mikaelian , Laryngoscope 1979, 89, 1. 10.1288/00005537-197901000-00001 423642

[17] E. M. Keithley , C. Canto , Q. Y. Zheng , N. Fischel‐Ghodsian , K. R. Johnson , Hear. Res. 2004, 188, 21. 14759567 10.1016/S0378-5955(03)00365-4 PMC2858220

[18] C. Lopez‐Otin , M. A. Blasco , L. Partridge , M. Serrano , G. Kroemer , Cell 2023, 186, 243. 36599349 10.1016/j.cell.2022.11.001

[19] Y. Zhang , X. Fu , Y. Li , W. Li , G. Hong , S. Guo , Y. Xiao , Z. Liu , S. Ding , X. Bi , F. Ye , J. Jin , R. Chai , Hum. Mol. Genet. 2023, 32, 1137. 36331344 10.1093/hmg/ddac270

[20] T. Yang , J. Kersigo , I. Jahan , N. Pan , B. Fritzsch , Hear. Res. 2011, 278, 21. 21414397 10.1016/j.heares.2011.03.002 PMC3130837

[21] S. Sun , T. Babola , G. Pregernig , K. S. So , M. Nguyen , S. M. Su , A. T. Palermo , D. E. Bergles , J. C. Burns , U. Muller , Cell 2018, 174, 1247. 30078710 10.1016/j.cell.2018.07.008 PMC6429032

[22] X. Fu , X. Sun , L. Zhang , Y. Jin , R. Chai , L. Yang , A. Zhang , X. Liu , X. Bai , J. Li , H. Wang , J. Gao , J. Clin. Invest. 2018, 128, 4938. 30247156 10.1172/JCI98058 PMC6205401

[23] X. Fu , P. Li , L. Zhang , Y. Song , Y. An , A. Zhang , W. Liu , C. Ye , Y. Zhang , R. Yue , X. Sun , R. Chai , H. Wang , J. Gao , Proc. Natl. Acad. Sci. USA 2022, 119, 2107357119. 10.1073/pnas.2107357119 PMC8917383 35238644

[24] A. Herranen , K. Ikaheimo , T. Lankinen , E. Pakarinen , B. Fritzsch , M. Saarma , M. Lindahl , U. Pirvola , Cell Death Dis. 2020, 11, 100. 32029702 10.1038/s41419-020-2286-6 PMC7005028

[25] P. Picciotti , A. Torsello , F. I. Wolf , G. Paludetti , E. Gaetani , R. Pola , Exp. Gerontol. 2004, 39, 1253. 15288700 10.1016/j.exger.2004.06.003

[26] A. R. Fetoni , A. Ferraresi , P. Picciotti , E. Gaetani , G. Paludetti , D. Troiani , Int. J. Audiol. 2009, 48, 804. 19951148 10.3109/14992020903023140

[27] L. I. Hellstrom , R. A. Schmiedt , Hear. Res. 1990, 50, 163. 2076969 10.1016/0378-5955(90)90042-n

[28] N. Li , W. Ma , F. Ren , X. Li , F. Li , W. Zong , L. Wu , Z. Dai , S. C. N. Hui , R. A. E. Edden , M. Li , F. Gao , Neuroimage 2023, 268, 119861. 36610677 10.1016/j.neuroimage.2023.119861 PMC10026366

[29] H. Lv , Z. Gao , Y. Wang , Y. Xie , M. Guan , H. Liao , Y. Xu , Am. J. Transl. Res. 2023, 15, 2407. 37193136 PMC10182487

[30] M. J. Manrique , A. Batuecas , C. Cenjor , S. Ferran , J. R. Gomez , A. I. Lorenzo , J. Marco , E. Matino , A. Morant , C. Morera , N. Perez , R. Polo , A. Ramos , S. Sanchez , F. Nunez , Acta Otorrinolaringol. Esp. (Engl Ed). 2023, 74, 124. 36906066 10.1016/j.otoeng.2023.03.002

[31] G. K. Picard , A. C. Bentvelzen , G. Savage , A. Barnier , P. A. Strutt , Ear Hear. 2023, 45, 297. 37635275 10.1097/AUD.0000000000001424

[32] G. Bredberg , Acta Otolaryngol. 1968, 236, 1. 4886545

[33] R. A. Altschuler , D. F. Dolan , K. Halsey , A. Kanicki , N. Deng , C. Martin , J. Eberle , D. C. Kohrman , R. A. Miller , J. Schacht , Neuroscience 2015, 292, 22. 25665752 10.1016/j.neuroscience.2015.01.068 PMC4511166

[34] J. R. Engle , S. Tinling , G. H. Recanzone , PLoS One. 2013, 8, 55092. 10.1371/journal.pone.0055092 PMC3563598 23390514

[35] K. K. Ohlemiller , A. R. Dahl , P. M. Gagnon , J. Assoc. Res. Otolaryngol. 2010, 11, 605. 20706857 10.1007/s10162-010-0228-1 PMC2975886

[36] L. M. Viana , J. T. O'Malley , B. J. Burgess , D. D. Jones , C. A. Oliveira , F. Santos , S. N. Merchant , L. D. Liberman , M. C. Liberman , Hear Res. 2015, 327, 78. 26002688 10.1016/j.heares.2015.04.014 PMC4554812

[37] I. Angelidis , L. M. Simon , I. E. Fernandez , M. Strunz , C. H. Mayr , F. R. Greiffo , G. Tsitsiridis , M. Ansari , E. Graf , T. M. Strom , M. Nagendran , T. Desai , O. Eickelberg , M. Mann , F. J. Theis , H. B. Schiller , Nat. Commun. 2019, 10, 963. 30814501 10.1038/s41467-019-08831-9 PMC6393476

[38] C. Tabula Muris , Nature 2020, 583, 590.32669714

[39] S. X. Leng , G. Pawelec , Life Med. 2022, 1, 67. 36699943 10.1093/lifemedi/lnac013 PMC9869752

[40] H. Zhang , J. Li , J. Ren , S. Sun , S. Ma , W. Zhang , Y. Yu , Y. Cai , K. Yan , W. Li , B. Hu , P. Chan , G. G. Zhao , J. C. I. Belmonte , Q. Zhou , J. Qu , S. Wang , G. H. Liu , Protein Cell. 2021, 12, 695. 34052996 10.1007/s13238-021-00852-9 PMC8403220

[41] T. Kusunoki , S. Cureoglu , P. A. Schachern , K. Baba , S. Kariya , M. M. Paparella , Otolaryngol. Head Neck Surg. 2004, 131, 897. 15577787 10.1016/j.otohns.2004.05.022

[42] M. A. Gratton , B. A. Schulte , Hear. Res. 1995, 82, 44. 7744712 10.1016/0378-5955(94)00161-i

[43] N. Sakaguchi , S. S. Spicer , G. N. Thomopoulos , B. A. Schulte , Hear. Res. 1997, 105, 44. 9083803 10.1016/s0378-5955(96)00180-3

[44] H. Liu , K. P. Giffen , L. Chen , H. J. Henderson , T. A. Cao , G. A. Kozeny , K. W. Beisel , Y. Li , D. Z. He , Cell Rep. 2022, 39, 110665. 35417713 10.1016/j.celrep.2022.110665 PMC9069708

[45] G. Sun , Y. Zheng , X. Fu , W. Zhang , J. Ren , S. Ma , S. Sun , X. He , Q. Wang , Z. Ji , F. Cheng , K. Yan , Z. Liu , J. C. I. Belmonte , J. Qu , S. Wang , R. Chai , G. H. Liu , Protein Cell. 2023, 14, 180. 36933008 10.1093/procel/pwac058 PMC10098046

[46] K. L. Elliott , B. Fritzsch , E. N. Yamoah , A. Zine , Front. Aging Neurosci. 2022, 14, 814528. 35250542 10.3389/fnagi.2022.814528 PMC8891613

[47] P. Wangemann , J. Physiol. 2006, 576, 11. 16857713 10.1113/jphysiol.2006.112888 PMC1995626

[48] I. Y. Y. Szeto , D. K. H. Chu , P. Chen , K. C. Chu , T. Y. K. Au , K. K. H. Leung , Y. H. Huang , S. L. Wynn , A. C. Y. Mak , Y. S. Chan , W. Y. Chan , R. Jauch , B. Fritzsch , M. H. Sham , R. Lovell‐Badge , K. S. E. Cheah , Proc. Natl. Acad. Sci. USA 2022, 119, 2122121119. 10.1073/pnas.2122121119 PMC9674217 36343245

[49] L. M. Friedman , A. A. Dror , K. B. Avraham , Int. J. Dev. Biol. 2007, 51, 609. 17891721 10.1387/ijdb.072365lf

[50] T. Sagara , H. Furukawa , K. Makishima , S. Fujimoto , Hear. Res. 1995, 83, 121. 7607978 10.1016/0378-5955(94)00195-v

[51] H. Hibino , Y. Kurachi , Physiology (Bethesda). 2006, 21, 336. 16990454 10.1152/physiol.00023.2006

[52] J. M. Renauld , V. Khan , M. L. Basch , Front. Cell Dev. Biol. 2022, 10, 867153. 35372344 10.3389/fcell.2022.867153 PMC8964366

[53] S. I. Kitajiri , M. Furuse , K. Morita , Y. Saishin‐Kiuchi , H. Kido , J. Ito , S. Tsukita , Hear. Res. 2004, 187, 25. 14698084 10.1016/s0378-5955(03)00338-1

[54] H. Locher , J. C. de Groot , L. van Iperen , M. A. Huisman , J. H. Frijns , Dev. Neurobiol. 2015, 75, 1219. 25663387 10.1002/dneu.22279 PMC5024031

[55] B. A. Schulte , R. A. Schmiedt , Hear. Res. 1992, 61, 35. 1326507 10.1016/0378-5955(92)90034-k

[56] P. Wangemann , Hear. Res. 2002, 165, 1. 12031509 10.1016/s0378-5955(02)00279-4

[57] S. S. Gill , A. N. Salt , Hear. Res. 1997, 113, 191. 9387998 10.1016/s0378-5955(97)00141-x

[58] L. J. Skinner , V. Enee , M. Beurg , H. H. Jung , A. F. Ryan , A. Hafidi , J. M. Aran , D. Dulon , J. Neurophysiol. 2003, 90, 320. 12611976 10.1152/jn.01155.2002

[59] L. Ruttiger , M. Sausbier , U. Zimmermann , H. Winter , C. Braig , J. Engel , M. Knirsch , C. Arntz , P. Langer , B. Hirt , M. Muller , I. Kopschall , M. Pfister , S. Munkner , K. Rohbock , I. Pfaff , A. Rusch , P. Ruth , M. Knipper , Proc. Natl. Acad. Sci. USA 2004, 101, 12922. 15328414 10.1073/pnas.0402660101 PMC516466

[60] Y. Wang , E. Fallah , E. S. Olson , Biophys. J. 2019, 116, 1769. 30992124 10.1016/j.bpj.2019.03.020 PMC6506630

[61] C. Kaur , P. Z. Wu , J. T. O'Malley , M. C. Liberman , J. Neurosci. 2023, 43, 8801. 37863653 10.1523/JNEUROSCI.1138-23.2023 PMC10727192

[62] G. Von Bekesy , Nature. 1952, 169, 241. 14910737 10.1038/169241a0

[63] B. Ding , J. P. Walton , X. Zhu , R. D. Frisina , Hear. Res. 2018, 367, 59. 30029086 10.1016/j.heares.2018.07.006 PMC7012304

[64] T. Liu , G. Li , K. V. Noble , Y. Li , J. L. Barth , B. A. Schulte , H. Lang , Neurobiol. Aging 2019, 80, 210. 31220650 10.1016/j.neurobiolaging.2019.04.009 PMC6679794

[65] Y. Zhou , J. Song , Y. P. Wang , A. M. Zhang , C. Y. Tan , Y. H. Liu , Z. P. Zhang , Y. Wang , K. T. Ma , L. Li , J. Q. Si , Mol. Med. Rep. 2019, 20, 1593. 31257512 10.3892/mmr.2019.10423 PMC6625423

[66] P. Z. Wu , J. T. O'Malley , V. de Gruttola , M. C. Liberman , J. Neurosci. 2020, 40, 6357. 32690619 10.1523/JNEUROSCI.0937-20.2020 PMC7424870

[67] R. D. Silva , M. R. F. Souza , A. S. B. Oliveira , M. C. M. Iorio , Codas 2021, 33, 20200021. 10.1590/2317-1782/20202020021 34406262

[68] J. Elander , E. M. McCormick , M. Varendh , K. Stenfeldt , R. D. Ganetzky , A. Goldstein , Z. Zolkipli‐Cunningham , L. E. MacMullen , R. Xiao , M. J. Falk , J. K. Ehinger , Mol. Genet. Metab. 2022, 137, 230. 36182714 10.1016/j.ymgme.2022.09.002 PMC9881581

[69] C. M. A. van Kempen , A. J. Beynon , J. J. Smits , M. C. H. Janssen , Mol. Genet. Metab. 2022, 135, 333. 35190254 10.1016/j.ymgme.2022.02.003

[70] T. Zou , B. Ye , K. Chen , A. Zhang , D. Guo , Y. Pan , R. Ding , H. Hu , X. Sun , M. Xiang , Front. Neurosci. 2022, 16, 998507. 36278017 10.3389/fnins.2022.998507 PMC9579438

[71] W. J. T. Tan , L. Song , M. Graham , A. Schettino , D. Navaratnam , W. G. Yarbrough , J. Santos‐Sacchi , A. V. Ivanova , Antioxid. Redox Signal. 2017, 27, 489. 28135838 10.1089/ars.2016.6851 PMC5564041

[72] H. Yamane , Y. Nakai , M. Takayama , H. Iguchi , T. Nakagawa , A. Kojima , Eur. Arch. Otorhinolaryngol. 1995, 252, 504. 8719596 10.1007/BF02114761

[73] K. K. Ohlemiller , J. S. Wright , L. L. Dugan , Audiol. Neurootol. 1999, 4, 229. 10436315 10.1159/000013846

[74] Y. Ohinata , J. M. Miller , R. A. Altschuler , J. Schacht , Brain Res. 2000, 878, 163. 10996147 10.1016/s0006-8993(00)02733-5

[75] K. K. Ohlemiller , M. E. R. Rice , J. M. Lett , P. M. Gagnon , Hear. Res. 2009, 249, 1. 19141317 10.1016/j.heares.2008.12.005

[76] D. C. Marcus , T. Wu , P. Wangemann , P. Kofuji , Am. J. Physiol. Cell Physiol. 2002, 282, C403. 11788352 10.1152/ajpcell.00312.2001

[77] W. F. Sewell , Hear. Res. 1984, 14, 305. 6480516 10.1016/0378-5955(84)90057-1

[78] R. A. Schmiedt , H. Lang , H. O. Okamura , B. A. Schulte , J. Neurosci. 2002, 22, 9643. 12417690 10.1523/JNEUROSCI.22-21-09643.2002 PMC6758027

[79] E. Bas , M. R. Anwar , T. R. Van De Water , Anat. Rec. (Hoboken) 2020, 303, 608. 30632705 10.1002/ar.24064

[80] Z. Zou , X. Long , Q. Zhao , Y. Zheng , M. Song , S. Ma , Y. Jing , S. Wang , Y. He , C. R. Esteban , N. Yu , J. Huang , P. Chan , T. Chen , J. C. Izpisua Belmonte , W. Zhang , J. Qu , G. H. Liu , Dev. Cell. 2021, 56, 383. 33238152 10.1016/j.devcel.2020.11.002

[81] H. J. Kim , M. A. Gratton , J. H. Lee , M. C. Perez Flores , W. Wang , K. J. Doyle , F. Beermann , M. A. Crognale , E. N. Yamoah , J. Neurosci. 2013, 33, 14601. 24005310 10.1523/JNEUROSCI.2147-13.2013 PMC3761058

[82] H. B. Zhao , BMC Cell Biol. 2016, 17, 16.27229462

[83] N. L. Samaha , M. M. Almasri , J. D. Johns , M. Hoa , Curr. Opin. Otolaryngol. Head Neck Surg. 2021, 29, 373. 34459799 10.1097/MOO.0000000000000738 PMC9047557

[84] A. C. Meyer , T. Moser , Curr. Opin. Otolaryngol. Head Neck Surg. 2010, 18, 441. 20802334 10.1097/MOO.0b013e32833e0586

[85] C. A. Makary , J. Shin , S. G. Kujawa , M. C. Liberman , S. N. Merchant , J. Assoc. Res. Otolaryngol. 2011, 12, 711. 21748533 10.1007/s10162-011-0283-2 PMC3214241

[86] L. G. Johnsson , J. E. Hawkins Jr. , Ann. Otol. Rhinol. Laryngol. 1972, 81, 179.4554886

[87] P. Z. Wu , L. D. Liberman , K. Bennett , V. de Gruttola , J. T. O'Malley , M. C. Liberman , Neuroscience 2019, 407, 8. 30099118 10.1016/j.neuroscience.2018.07.053 PMC6369025

[88] E. M. Keithley , J. Neurosci. Res. 2020, 98, 1674. 31066107 10.1002/jnr.24439 PMC7496655

[89] A. Wright , A. Davis , G. Bredberg , L. Ulehlová , H. Spencer , G. Bock , H. Felix , S. Iurato , L. G. Johnsson , M. Pauler , Acta Otolaryngol. Suppl 1987, 436, 15. 3478958 10.3109/00016488709124972

[90] E. Felder , A. Schrott‐Fischer , Hear. Res. 1995, 91, 19. 8647720 10.1016/0378-5955(95)00158-1

[91] Y. Sergeyenko , K. Lall , M. C. Liberman , S. G. Kujawa , J. Neurosci. 2013, 33, 13686. 23966690 10.1523/JNEUROSCI.1783-13.2013 PMC3755715

[92] S. Stamataki , H. W. Francis , M. Lehar , B. J. May , D. K. Ryugo , Hear. Res. 2006, 221, 104. 17005343 10.1016/j.heares.2006.07.014

[93] K. A. Fernandez , P. W. Jeffers , K. Lall , M. C. Liberman , S. G. Kujawa , J. Neurosci. 2015, 35, 7509. 25972177 10.1523/JNEUROSCI.5138-14.2015 PMC4429155

[94] E. Lobarinas , R. Salvi , D. Ding , Hear. Res. 2013, 302, 113. 23566980 10.1016/j.heares.2013.03.012 PMC3695223

[95] E. Lobarinas , R. Salvi , D. Ding , J. Assoc. Res. Otolaryngol. 2016, 17, 89. 26691159 10.1007/s10162-015-0550-8 PMC4791417

[96] R. A. Friedman , L. Van Laer , M. J. Huentelman , S. S. Sheth , E. Van Eyken , J. J. Corneveaux , W. D. Tembe , R. F. Halperin , A. Q. Thorburn , S. Thys , S. Bonneux , E. Fransen , J. Huyghe , I. Pyykko , C. W. Cremers , H. Kremer , I. Dhooge , D. Stephens , E. Orzan , M. Pfister , M. Bille , A. Parving , M. Sorri , P. H. Van de Heyning , L. Makmura , J. D. Ohmen , F. H. Linthicum Jr. , J. N. Fayad , J. V. Pearson , D. W. Craig , et al., Hum. Mol. Genet. 2009, 18, 785. 19047183 10.1093/hmg/ddn402 PMC2638831

[97] L. Van Laer , J. R. Huyghe , S. Hannula , E. Van Eyken , S. DA , E. Maki‐Torkko , P. Aikio , E. Fransen , A. Lysholm‐Bernacchi , M. Sorri , M. J. Huentelman , G. Van Camp , Eur. J. Hum. Genet. 2010, 18, 685. 20068591 10.1038/ejhg.2009.234 PMC2987344

[98] D. L. Newman , L. M. Fisher , J. Ohmen , R. Parody , C. T. Fong , S. T. Frisina , F. Mapes , D. A. Eddins , D. Robert Frisina , R. D. Frisina , R. A. Friedman , Hear. Res. 2012, 294, 125. 23102807 10.1016/j.heares.2012.08.016 PMC3705704

[99] H. Luo , T. Yang , X. Jin , X. Pang , J. Li , Y. Chai , L. Li , Y. Zhang , L. Zhang , Z. Zhang , W. Wu , Q. Zhang , X. Hu , J. Sun , X. Jiang , Z. Fan , Z. Huang , H. Wu , PLoS One. 2013, 8, 77153. 10.1371/journal.pone.0077153 PMC3795658 24146964

[100] T. J. Hoffmann , B. J. Keats , N. Yoshikawa , C. Schaefer , N. Risch , L. R. Lustig , PLoS Genet. 2016, 12, 1006371. 10.1371/journal.pgen.1006371 PMC5072625 27764096

[101] S. Kitajiri , T. Sakamoto , I. A. Belyantseva , R. J. Goodyear , R. Stepanyan , I. Fujiwara , J. E. Bird , S. Riazuddin , S. Riazuddin , Z. M. Ahmed , J. E. Hinshaw , J. Sellers , J. R. Bartles , J. A. Hammer 3rd , G. P. Richardson , A. J. Griffith , G. I. Frolenkov , T. B. Friedman , Cell. 2010, 141, 786. 20510926 10.1016/j.cell.2010.03.049 PMC2879707

[102] A. Forge , J. Schacht , A. antibiotics , Audiol. Neurootol. 2000, 5, 3. 10686428 10.1159/000013861

[103] S. Nyberg , N. J. Abbott , X. Shi , P. S. Steyger , A. Dabdoub , Sci. Transl. Med. 2019, 11, aa0935. 10.1126/scitranslmed.aao0935 PMC6488020 30842313

[104] J. Wang , R. V. Lloyd Faulconbridge , A. Fetoni , M. J. Guitton , R. Pujol , J. L. Puel , Neuropharmacology 2003, 45, 380. 12871655 10.1016/s0028-3908(03)00194-1

[105] Y. Joo , K. J. Cruickshanks , B. E. K. Klein , R. Klein , O. Hong , M. I. Wallhagen , J. Gerontol. A Biol. Sci. Med. Sci. 2020, 75, 561. 31282945 10.1093/gerona/glz166 PMC7328195

[106] N. Fischel‐Ghodsian , Y. Bykhovskaya , K. Taylor , T. Kahen , R. Cantor , K. Ehrenman , R. Smith , E. Keithley , Hear. Res. 1997, 110, 147. 9282897 10.1016/s0378-5955(97)00077-4

[107] U. Bai , M. D. Seidman , R. Hinojosa , W. S. Quirk , Am. J. Otol. 1997, 18, 449. 9233484

[108] A. Markaryan , E. G. Nelson , R. Hinojosa , Mutat. Res. 2008, 640, 38. 18242646 10.1016/j.mrfmmm.2007.12.007

[109] A. R. Lyu , T. H. Kim , S. J. Park , S. A. Shin , S. H. Jeong , Y. Yu , Y. H. Huh , A. R. Je , M. J. Park , Y. H. Park , Int. J. Mol. Sci. 2020, 21, 2505. 32260310 10.3390/ijms21072505 PMC7177801

[110] G. Perkins , J. H. Lee , S. Park , M. Kang , M. C. Perez‐Flores , S. Ju , G. Phillips , A. Lysakowski , M. A. Gratton , E. N. Yamoah , J. Neurosci. 2020, 40, 8556. 33020216 10.1523/JNEUROSCI.2901-19.2020 PMC7605424

[111] S. Hashimoto , H. F. Schuknecht , Ann. Otol. Rhinol. Laryngol. 1987, 96, 229. 3566065 10.1177/000348948709600219

[112] F. Zhang , M. Dai , L. Neng , J. H. Zhang , Z. Zhi , A. Fridberger , X. Shi , FASEB J. 2013, 27, 3730. 23729595 10.1096/fj.13-232892 PMC3752533

[113] C. Petitpré , H. Wu , A. Sharma , A. Tokarska , P. Fontanet , Y. Wang , F. Helmbacher , K. Yackle , G. Silberberg , S. Hadjab , F. Lallemend , Nat. Commun. 2018, 9, 3691. 30209249 10.1038/s41467-018-06033-3 PMC6135759

[114] K. L. Elliott , J. Kersigo , J. H. Lee , I. Jahan , G. Pavlinkova , B. Fritzsch , E. N. Yamoah , Front. Cell. Neurosci. 2021, 15, 678113. 34211371 10.3389/fncel.2021.678113 PMC8239239

[115] N. Y. Kiang , M. B. Sachs , W. T. Peake , J. Acoust. Soc. Am. 1967, 42, 1341. 6081601 10.1121/1.1910723

[116] E. N. Flores , A. Duggan , T. Madathany , A. K. Hogan , F. G. Márquez , G. Kumar , R. P. Seal , R. H. Edwards , M. C. Liberman , J. García‐Añoveros , Curr. Biol. 2015, 25, 606. 25639244 10.1016/j.cub.2015.01.009 PMC4348215

[117] M. Barclay , A. F. Ryan , G. D. Housley , Neural Dev. 2011, 6, 33. 21989106 10.1186/1749-8104-6-33 PMC3207869

[118] S. Sun , T. Babola , G. Pregernig , K. S. So , M. Nguyen , S. M. Su , A. T. Palermo , D. E. Bergles , J. C. Burns , U. Müller , Cell 2018, 174, 1247. 30078710 10.1016/j.cell.2018.07.008 PMC6429032

[119] M. Wang , S. Lin , R. Xie , PLoS One 2023, 18, 0292676. 10.1371/journal.pone.0292676 PMC10602254 37883357

[120] B. R. Shrestha , C. Chia , L. Wu , S. G. Kujawa , M. C. Liberman , L. V. Goodrich , Cell 2018, 174, 1229. 30078709 10.1016/j.cell.2018.07.007 PMC6150604

[121] S. G. Kujawa , M. C. Liberman , J. Neurosci. 2009, 29, 14077. 19906956 10.1523/JNEUROSCI.2845-09.2009 PMC2812055

[122] Y. Zilberstein , M. C. Liberman , G. Corfas , J. Neurosci. 2012, 32, 405. 22238076 10.1523/JNEUROSCI.4678-11.2012 PMC3678770

[123] J. Kersigo , B. Fritzsch , Front. Aging Neurosci. 2015, 7, 00033. 10.3389/fnagi.2015.00033 PMC4364252 25852547

[124] H. Felix , L. G. Johnsson , M. Gleeson , A. Pollak , Acta Otolaryngol. 1990, 470, 71. 10.3109/00016488909138359 2239237

[125] M. A. Chen , P. Webster , E. Yang , F. H. Linthicum Jr. , Otol. Neurotol. 2006, 27, 316. 16639268 10.1097/00129492-200604000-00005

[126] J. B. Nadol Jr. , Otolaryngol. Head Neck Surg. 1979, 87, 818. 530702 10.1177/019459987908700617

[127] K. Hadrian , H. Melkonyan , S. Schlatt , J. Wistuba , S. Wasmuth , A. Heiligenhaus , S. Thanos , M. R. R. Bohm , Exp. Eye Res. 2019, 185, 107676. 31128101 10.1016/j.exer.2019.05.016

[128] M. Shimobayashi , M. N. Hall , Nat. Rev. Mol. Cell. Biol. 2014, 15, 155. 24556838 10.1038/nrm3757

[129] M. Laplante , D. M. Sabatini , Cell 2012, 149, 274. 22500797 10.1016/j.cell.2012.03.017 PMC3331679

[130] X. Bai , Y. Jiang , Cell. Mol. Life Sci. 2010, 67, 239. 19823764 10.1007/s00018-009-0163-7 PMC4780839

[131] C. C. Dibble , L. C. Cantley , Trends Cell Biol. 2015, 25, 545. 26159692 10.1016/j.tcb.2015.06.002 PMC4734635

[132] B. D. Manning , A. Toker , Cell 2017, 169, 381. 28431241 10.1016/j.cell.2017.04.001 PMC5546324

[133] R. A. Altschuler , A. Kanicki , C. Martin , D. C. Kohrman , R. A. Miller , Hear. Res. 2018, 370, 11. 30245283 10.1016/j.heares.2018.09.003 PMC6240471

[134] R. A. Altschuler , L. Kabara , C. Martin , A. Kanicki , C. E. Stewart , D. C. Kohrman , D. F. Dolan , Front. Cell Neurosci. 2021, 15, 658972. 33897373 10.3389/fncel.2021.658972 PMC8058174

[135] A. González , M. N. Hall , S. C. Lin , D. G. Hardie , Cell Metab. 2020, 31, 472. 32130880 10.1016/j.cmet.2020.01.015

[136] S. G. Rhee , S. H. Bae , Free Radical Biol. Med. 2015, 88, 205. 26117317 10.1016/j.freeradbiomed.2015.06.007

[137] C. Zhang , W. Sun , J. Li , B. Xiong , M. D. Frye , D. Ding , R. Salvi , M. J. Kim , S. Someya , B. H. Hu , Neuroscience 2017, 361, 179. 28818524 10.1016/j.neuroscience.2017.08.015 PMC5605466

[138] C. Zhao , Z. Yang , Z. Chen , W. Liang , S. Gong , Z. Du , Mol. Med. 2022, 28, 124. 36266633 10.1186/s10020-022-00552-y PMC9583487

[139] J. Zhao , G. Li , X. Zhao , X. Lin , Y. Gao , N. Raimundo , G. L. Li , W. Shang , H. Wu , L. Song , Aging (Albany NY). 2020, 12, 5590. 32240104 10.18632/aging.102977 PMC7185105

[140] C. Chen , M. Zhou , Y. Ge , X. Wang , Mech. Ageing Dev. 2020, 187, 111215. 32084459 10.1016/j.mad.2020.111215

[141] C. Cantó , J. Auwerx , Curr. Opin. Lipidol. 2009, 20, 98. 19276888 10.1097/MOL.0b013e328328d0a4 PMC3627054

[142] H. Xiong , M. Dai , Y. Ou , J. Pang , H. Yang , Q. Huang , S. Chen , Z. Zhang , Y. Xu , Y. Cai , M. Liang , X. Zhang , L. Lai , Y. Zheng , Exp Gerontol. 2014, 51, 8. 24365660 10.1016/j.exger.2013.12.006

[143] H. Xiong , J. Pang , H. Yang , M. Dai , Y. Liu , Y. Ou , Q. Huang , S. Chen , Z. Zhang , Y. Xu , L. Lai , Y. Zheng , Neurobiol. Aging 2015, 36, 1692. 25638533 10.1016/j.neurobiolaging.2014.12.034

[144] H. Xiong , S. Chen , L. Lai , H. Yang , Y. Xu , J. Pang , Z. Su , H. Lin , Y. Zheng , Neurobiol. Aging 2019, 79, 30. 31026620 10.1016/j.neurobiolaging.2019.03.013

[145] T. Xue , L. Wei , D. J. Zha , J. H. Qiu , F. Q. Chen , L. Qiao , Y. Qiu , Int. J. Mol. Med. 2016, 38, 1387. 27635430 10.3892/ijmm.2016.2735 PMC5065299

[146] S. Hao , L. Wang , K. Zhao , X. Zhu , F. Ye , J. Cell Biochem. 2019, 120, 4975. 30556210 10.1002/jcb.27773

[147] W. Xie , T. Shu , H. Peng , J. Liu , C. Li , M. Wang , P. Wu , Y. Liu , Acta Biochim. Biophys. Sin (Shanghai). 2022, 54, 332. 35538041 10.3724/abbs.2022018 PMC9828013

[148] J. Pang , H. Xiong , Y. Ou , H. Yang , Y. Xu , S. Chen , L. Lai , Y. Ye , Z. Su , H. Lin , Q. Huang , X. Xu , Y. Zheng , Neurobiol. Aging 2019, 80, 127. 31170533 10.1016/j.neurobiolaging.2019.04.003

[149] H. J. Kim , W. Cao , G. S. Oh , S. Lee , A. Shen , D. Khadka , S. B. Lee , S. Sharma , S. Y. Kim , S. K. Choe , T. H. Kwak , J. M. Kim , R. Park , H. S. So , Aging Cell. 2019, 18, 13016. 10.1111/acel.13016 PMC6718544 31353811

[150] S. A. Salam , F. Mostafa , M. M. Alnamshan , S. S. Elshewemi , J. M. Sorour , Biomed. Pharmacother. 2021, 143, 112149. 34507120 10.1016/j.biopha.2021.112149

[151] G. Tian , J. Sawashita , H. Kubo , S. Y. Nishio , S. Hashimoto , N. Suzuki , H. Yoshimura , M. Tsuruoka , Y. Wang , Y. Liu , H. Luo , Z. Xu , M. Mori , M. Kitano , K. Hosoe , T. Takeda , S. Usami , K. Higuchi , Antioxid. Redox Signal. 2014, 20, 2606. 24124769 10.1089/ars.2013.5406 PMC4025630

[152] C. Han , P. Linser , H. J. Park , M. J. Kim , K. White , J. M. Vann , D. Ding , T. A. Prolla , S. Someya , Neurobiol. Aging 2016, 43, 58. 27255815 10.1016/j.neurobiolaging.2016.03.023 PMC4893170

[153] S. Someya , W. Yu , W. C. Hallows , J. Xu , J. M. Vann , C. Leeuwenburgh , M. Tanokura , J. M. Denu , T. A. Prolla , Cell. 2010, 143, 802. 21094524 10.1016/j.cell.2010.10.002 PMC3018849

[154] Q. Ma , Annu. Rev. Pharmacol. Toxicol. 2013, 53, 401. 23294312 10.1146/annurev-pharmtox-011112-140320 PMC4680839

[155] A. R. Fetoni , V. Zorzi , F. Paciello , G. Ziraldo , C. Peres , M. Raspa , F. Scavizzi , A. M. Salvatore , G. Crispino , G. Tognola , G. Gentile , A. G. Spampinato , D. Cuccaro , M. Guarnaccia , G. Morello , G. Van Camp , E. Fransen , M. Brumat , G. Girotto , G. Paludetti , P. Gasparini , S. Cavallaro , F. Mammano , Redox Biol. 2018, 19, 301. 30199819 10.1016/j.redox.2018.08.002 PMC6129666

[156] H. Du , X. Zhou , L. Shi , M. Xia , Y. Wang , N. Guo , H. Hu , P. Zhang , H. Yang , F. Zhu , Z. Teng , C. Liu , M. Zhao , Front. Mol. Neurosci. 2022, 15, 829642. 35283722 10.3389/fnmol.2022.829642 PMC8908960

[157] J. K. Harrison , Y. Jiang , S. Chen , Y. Xia , D. Maciejewski , R. K. McNamara , W. J. Streit , M. N. Salafranca , S. Adhikari , D. A. Thompson , P. Botti , K. B. Bacon , L. Feng , Proc. Natl. Acad. Sci. USA 1998, 95, 10896. 9724801 10.1073/pnas.95.18.10896 PMC27992

[158] V. Julia , Allergy 2012, 67, 1106. 22765026 10.1111/j.1398-9995.2012.02870.x

[159] G. E. White , D. R. Greaves , Arterioscler., Thromb., Vasc. Biol. Mar 2012, 32, 589. 10.1161/ATVBAHA.111.237412 22247260

[160] A. R. Stothert , T. Kaur , Front. Cell. Neurosci. 2021, 15, 694292. 34408629 10.3389/fncel.2021.694292 PMC8365835

[161] T. Kaur , D. Zamani , L. Tong , E. W. Rubel , K. K. Ohlemiller , K. Hirose , M. E. Warchol , J. Neurosci. 2015, 35, 15050. 26558776 10.1523/JNEUROSCI.2325-15.2015 PMC4642237

[162] Z. Zhao , L. Ma , Proc. Natl. Acad. Sci. USA. 2009, 106, 18016. 19805191 10.1073/pnas.0906880106 PMC2764873

[163] J. L. Fitzakerley , G. J. Trachte , Physiol. Genom. 2018, 50, 780. 10.1152/physiolgenomics.00056.2018 29958079

[164] H. Furuta , N. Mori , L. Luo , A. F. Ryan , Hear. Res. 1995, 92, 78. 8647748 10.1016/0378-5955(95)00203-0

[165] F. Sun , K. Zhou , K. Y. Tian , X. Y. Zhang , W. Liu , J. Wang , C. P. Zhong , J. H. Qiu , D. J. Zha , Front. Cell Dev. Biol. 2021, 9, 681421. 34268307 10.3389/fcell.2021.681421 PMC8276373

[166] J. S. Stone , D. A. Cotanche , Int. J. Dev. Biol. 2007, 51, 633. 17891722 10.1387/ijdb.072408js

[167] J. Wang , J. L. Puel , Physiol. Rev. 2018, 98, 2477. 30156495 10.1152/physrev.00053.2017

[168] J. Qi , W. Huang , Y. Lu , X. Yang , Y. Zhou , T. Chen , X. Wang , Y. Yu , J. Q. Sun , R. Chai , Neurosci. Bull. 2023, 40, 113. 37787875 10.1007/s12264-023-01130-w PMC10774470

[169] Y. Tao , X. Liu , L. Yang , C. Chu , F. Tan , Z. Yu , J. Ke , X. Li , X. Zheng , X. Zhao , J. Qi , C. P. Lin , R. Chai , G. Zhong , H. Wu , Signal Transduction Targeted Ther. 2022, 7, 109. 10.1038/s41392-022-00938-8 PMC9023545 35449181

[170] E. N. Yamoah , M. Li , A. Shah , K. L. Elliott , K. Cheah , P. X. Xu , S. Phillips , S. M. Young , D. F. Eberl , B. Fritzsch , Ageing Res. Rev. 2020, 59, 101042. 32173536 10.1016/j.arr.2020.101042 PMC7261488

[171] J. Lv , H. Wang , X. T. Cheng , Y. X. Chen , D. Q. Wang , L. L. Zhang , Q. Cao , H. H. Tang , S. W. Hu , K. Y. Gao , M. Z. Xun , J. H. Wang , Z. J. Wang , B. Y. Zhu , C. Cui , Z. W. Gao , L. Guo , S. Yu , L. Y. Jiang , Y. B. Yin , J. J. Zhang , B. Chen , W. Q. Wang , R. J. Chai , Z. Y. Chen , H. W. Li , Y. L. Shu , Lancet 2024, 403, 2317. 38280389 10.1016/S0140-6736(23)02874-X

[172] J. Y. Qi , F. Z. Tan , L. Y. Zhang , L. Lu , S. Z. Zhang , Y. B. Zhai , Y. C. Lu , X. Y. Qian , W. X. Dong , Y. Y. Zhou , Z. Y. Zhang , X. H. Yang , L. L. Jiang , C. R. Yu , J. C. Liu , T. Chen , L. Q. Wu , C. Tan , S. J. Sun , H. Song , Y. L. Shu , L. Xu , X. Gao , H. W. Li , R. J. Chai , Adv. Sci. 2024, 11, 2306788. 10.1002/advs.202306788 PMC10953563 38189623

[173] S. Mukherjee , M. Kuroiwa , W. Oakden , B. T. Paul , A. Noman , J. Chen , V. Lin , A. Dimitrijevic , G. Stanisz , T. N. Le , Mol. Ther. 2022, 30, 519. 34298130 10.1016/j.ymthe.2021.07.013 PMC8821893

[174] H. Chen , Y. Xing , L. Xia , Z. Chen , S. Yin , J. Wang , Gene Ther. 2018, 25, 251. 29535374 10.1038/s41434-018-0012-0 PMC6062503

[175] M. Dermit , P. Casado , V. Rajeeve , E. H. Wilkes , D. E. Foxler , H. Campbell , S. Critchlow , T. V. Sharp , J. G. Gribben , R. Unwin , P. R. Cutillas , Oncogene 2017, 36, 2762. 27991931 10.1038/onc.2016.435 PMC5362070

[176] T. Nacarelli , A. Azar , C. Sell , Free Radical Biol. Med. 2016, 95, 133. 27016071 10.1016/j.freeradbiomed.2016.03.008

[177] D. E. Harrison , R. Strong , Z. D. Sharp , J. F. Nelson , C. M. Astle , K. Flurkey , N. L. Nadon , J. E. Wilkinson , K. Frenkel , C. S. Carter , M. Pahor , M. A. Javors , E. Fernandez , R. A. Miller , Nature 2009, 460, 392. 19587680 10.1038/nature08221 PMC2786175

[178] H. Liu , F. Li , X. Li , Q. Wu , C. Dai , Neurosci. Lett. 2022, 772, 136493. 35114332 10.1016/j.neulet.2022.136493

[179] O. S. Choo , Y. Y. Lee , Y. S. Kim , Y. J. Kim , D. H. Lee , H. Kim , J. H. Jang , Y. H. Choung , Bba‐Mol. Cell. Res. 2022, 1869, 119331. 10.1016/j.bbamcr.2022.119331 35963547

[180] J. Halonen , A. S. Hinton , R. D. Frisina , B. Ding , X. X. Zhu , J. P. Walton , Hear. Res. 2016, 336, 63. 27157488 10.1016/j.heares.2016.05.001 PMC7416673

[181] D. O. J. Reijntjes , S. J. Pyott , Hear. Res. 2016, 336, 1. 27018296 10.1016/j.heares.2016.03.011

[182] L. Bergstrom , P. Thompson , I. Sando , R. P. Wood , Surg. Forum 1976, 27, 532. 1019966

[183] Z. Jafari , B. E. Kolb , M. H. Mohajerani , Ageing Res. Rev. 2019, 56, 100963. 31557539 10.1016/j.arr.2019.100963

[184] Y. Z. Quan , W. Wei , V. Ergin , A. P. Rameshbabu , M. Huang , C. Tian , S. V. Saladi , A. A. Indzhykulian , Z. Y. Chen , Proc. Natl. Acad. Sci. USA 2023, 120, 2215253120. 10.1073/pnas.2215253120 PMC10151514 37068229

[185] K. R. Koehler , J. Nie , E. Longworth‐Mills , X. P. Liu , J. Lee , J. R. Holt , E. Hashino , Nat. Biotechnol. 2017, 35, 583. 28459451 10.1038/nbt.3840 PMC5462862

[186] P. J. Atkinson , A. K. Wise , B. O. Flynn , B. A. Nayagam , R. T. Richardson , PLoS One 2014, 9, 102077. 10.1371/journal.pone.0102077 PMC4103851 25036727

[187] M. Allocca , M. Doria , M. Petrillo , P. Colella , M. Garcia‐Hoyos , D. Gibbs , S. R. Kim , A. Maguire , T. S. Rex , U. Di Vicino , L. Cutillo , J. R. Sparrow , D. S. Williams , J. Bennett , A. Auricchio , J. Clin. Invest. 2008, 118, 1955. 18414684 10.1172/JCI34316 PMC2298836

